# The essence of *NAC* gene family to the cultivation of drought-resistant soybean *(Glycine max L. Merr.)* cultivars

**DOI:** 10.1186/s12870-017-1001-y

**Published:** 2017-02-28

**Authors:** Reem M. Hussain, Mohammed Ali, Xing Feng, Xia Li

**Affiliations:** 1 State Key Laboratory of Agricultural Microbiology, College of Plant Science and Technology, Huazhong Agricultural University, Wuhan, 430070 People’s Republic of China; 2 Tishreen University, Faculty of Agriculture, Crop Field Department, Tishreen University, Lattakia, Syria; 3 National Key Lab of Crop Genetic Improvement, College of Life science and Technology, Bioinformatics Lab, Huazhong Agriculture University, Wuhan, 430070 Hubei People’s Republic of China

**Keywords:** Drought stress, NAC transcription factor, Real-time quantitative PCR, Soybean (*Glycine max* [L.] Merr)

## Abstract

**Background:**

The *NAC* gene family is notable due to its large size, as well as its relevance in crop cultivation – particularly in terms of enhancing stress tolerance of plants. These plant-specific proteins contain NAC domain(s) that are named after Petunia NAM and Arabidopsis ATAF1/2 and CUC2 transcription factors based on the consensus sequence they have. Despite the knowledge available regarding NAC protein function, an extensive study on the possible use of *GmNACs* in developing soybean cultivars with superior drought tolerance is yet to be done.

**Results:**

In response to this, our study was carried out, mainly through means of phylogenetic analysis (rice and *Arabidopsis NAC* genes served as seeding sequences). Through this, 139 *GmNAC* genes were identified and later grouped into 17 clusters. Furthermore, real-time quantitative PCR was carried out on drought-stressed and unstressed leaf tissues of both sensitive (B217 and H228) and tolerant (Jindou 74 and 78) cultivars. This was done to analyze the gene expression of 28 dehydration-responsive *GmNAC* genes. Upon completing the analysis, it was found that *GmNAC* gene expression is actually dependent on genotype. Eight of the 28 selected genes (*GmNAC004, GmNAC021, GmNAC065, GmNAC066, GmNAC073, GmNAC082, GmNAC083* and *GmNAC087*) were discovered to have high expression levels in the drought-resistant soybean varieties tested. This holds true for both extreme and standard drought conditions. Alternatively, the drought-sensitive cultivars exhibited lower *GmNAC* expression levels in comparison to their tolerant counterparts.

**Conclusion:**

The study allowed for the identification of eight *GmNAC* genes that could be focused upon in future attempts to develop superior soybean varieties, particularly in terms of drought resistance. This study revealed that there were more dehydration-responsive *GmNAC* genes as (*GmNAC004, GmNAC005, GmNAC020* and *GmNAC021)* in addition to what were reported in earlier inquiries. It is important to note though, that discovering such notable genes is not the only goal of the study. It managed to put emphasis on the significance of further understanding the potential of soybean *GmNAC* genes, for the purpose of enhancing tolerance towards abiotic stress in general. This scientific inquiry has also revealed that cultivar genotypes tend to differ in their drought-induced gene expression.

**Electronic supplementary material:**

The online version of this article (doi:10.1186/s12870-017-1001-y) contains supplementary material, which is available to authorized users.

## Background

Being an excellent source of protein and calcium (surpassing other major staples such as wheat and sorghum), soybean (*Glycine max* [L.] Merr) is generally considered the most valuable legume variety. Aside from being produced for human consumption, the plant is used to feed livestock – hence further increasing its significance as a food source [1, 2]. Moreover, its oil is used in biodiesel production [2, 3]. In 2015 and 2016, soybean yield worldwide is estimated at 320.15 million metric tons [4].

Still, like other crops, soybean is normally exposed to a myriad of stress factors, ranging from the biotic (organism-induced) to the abiotic (environmental causes) [5]. Without proper management or sufficient tolerance, stresses can limit plant development and thus affect overall yield [6]. Among stress factors though, droughts brought forth by climate change have become among the most concerning, and are expected to adversely affect soybean production around the world in the years to come [6, 7]. Thus, it is important to choose cultivars with the highest drought tolerance to address this expected threat to proteins and food security [8].

The NAC transcription factor family is a big group of proteins, which are named after three important proteins, Petunia No Apical Meristem (NAM), Arabidopsis transcription activation factors (ATAF1 and ATAF2) and Cup-shaped cotyledon 2 (CUC2) [9, 10]. NAM is essential in the *Petunia* plant’s embryo development and flower pattern formation [11]. In *Arabidopsis*, *CUC2* is vital to embryo, flower, and apical meristem growth [12], while *ATAF1* and *ATAF2* are known to be responsive to stress factors [13–16]. The NAM domain content of these *NAC* genes covers N-terminus subdomains A to E [17, 18]. Subdomains B and E are flexible, while the rest are highly conserved [19].

Several plant species already have identified *NAC* gene numbers. Grape (*Vitis vinifera*) has 74 [20], pigeonpea (*Cajanus cajan*) has 88 [21], *Arabidopsis* has 117 [22], foxtail millet (*Setaria italica*) has 147 [23], rice (*Oryza sativa*) has 151 [22], soybean (*Glycine max*) has 152 [24], and Chinese cabbage (*Brassica pekinensis*) has 204 [25]. Based on studies, *NAC* genes are involved in various developmental processes in plants [10, 15]. These developmental processes encompass stress responses [19, 20], hormone signaling [18], fruit ripening [17], leaf senescence [26], organ formation and development [27], and apical meristem development [10, 15].

NAC transcription factors are relevant in both abscisic acid (ABA) dependent and independent pathways in drought stress signaling [28, 29]. This was first revealed in the discovery of multiple stress-responsive genes in *Arabidopsis*, namely *ANAC019, GmNAC055*, and *GmNAC072*. The overexpression of the aforementioned genes led to enhanced drought tolerance [30, 31]. In rice, one notable *NAC* gene is *SNAC1*, which is vital to drought stress signaling in guard cells. The overexpression of *SNAC1* improves the drought tolerance of transgenic varieties [32]. *OsNAC10*, a root-specific transcription factor, is another noteworthy *NAC*. Overexpression of this gene enhances drought tolerance and provides another valuable benefit – increased grain production (under normal circumstances) [33]. *SNAC2/OsNAC6, Os045,* and *Os063* are other genes in rice whose overexpression boosts tolerance to abiotic stress factors (such as salinity, drought and cold) [34–37].

Shifting focus towards soybean, Meng et al. [38] recognized the first six *GmNAC* genes (designated 1 to 6). The expressions of *GmNAC1-6* under osmotic stress were examined [39]. A more comprehensive research on *GmNAC* genes in soybean was conducted later on. In the said study, 9 of the 31 *GmNAC* genes tested were affected by abiotic stress factors, such as ABA treatments, high salinity, cold, and/or dehydration [40].

In 2011, Le et al. carried out another inquiry on soybean *GmNAC* genes, in which 152 full-length *GmNAC* transcription factors (inclusive of 11 membrane-bound transcription factors) were identified [23]. The researchers also discovered that out of the 38 *GmNAC* genes tested, six were repressed and 25 induced twice as much or more upon being subjected to dehydration treatment under real-time quantitative PCR (RT-qPCR) [23]. The same group of *GmNAC* genes also showed differences in drought-responsive expressions within the same tissue, at different developmental stages, and in different tissues at the same developmental stage [41].

Due to the recognized role of NAC transcription factors in various physiological and biological processes, a more comprehensive study of the *NAC* gene family in soybean was done. This was to establish the possible application of the said genes in creating highly drought-tolerant transgenic cultivars. The research involved a total of 28 *GmNAC* genes from drought-sensitive (B217 and H228) and drought-tolerant (Jindou74 and 78) soybean varieties. It was hypothesized that the varying expression of *GmNAC* genes might have an impact on drought tolerance. Furthermore, the possible correlation between *GmNACs* and tolerance degrees was looked into.

## Results and discussions

### Identification of NAC Transcription factors and phylogenetic analysis of *NAC* genes in soybean, *Arabidopsis,* and rice

NAC transcription factors in soybean were identified using Phytozome v.10.2 database using BLAST search technique and two well-known NAC proteins from rice and *Arabidopsis* were used as query sequences. During the search, the presence of NAC domain was found and confirmed and furthermore redundant sequences were removed. Non-redundant putative *NAC* genes which were identified in soybean genome were 139 *GmNACs*.

From the alignments of predicted NAC proteins, phylogenetic tree was constructed and *GmNAC001* to *GmNAC139* were designated in accordance with the tree. These listings are shown in Additional file 1. In these *GmNACs* genes, the coding proteins were composed of 190 to 378 amino acids in length. For every orthologous which is found in rice and *Arabidopsis*, two or more soybean *NAC* genes were found in most of the cases. Additional file 2 contains detailed information along with accession numbers regarding *NAC* family genes present in rice and *Arabidopsis*.

**Fig. 1:**
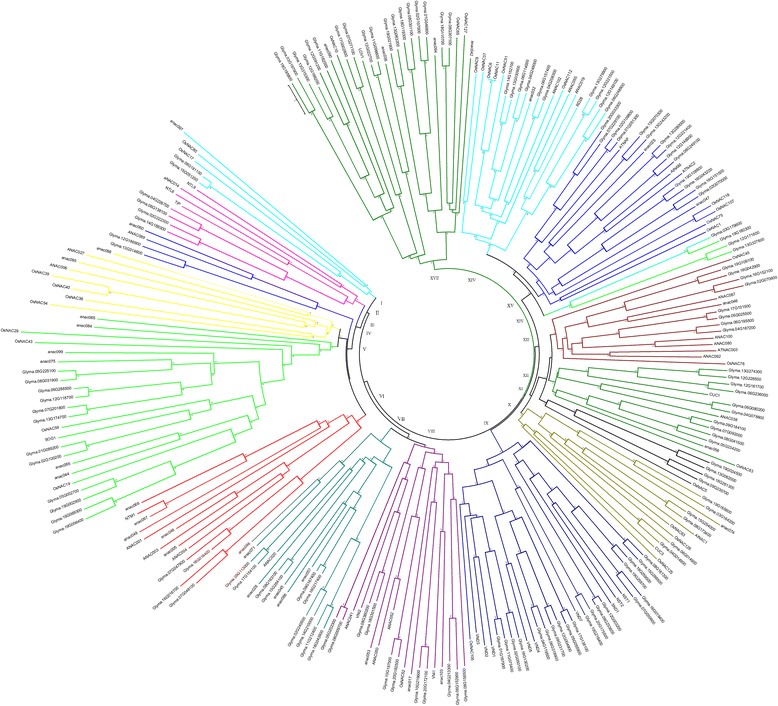
Phylogenetic analysis of NAC proteins from *Arabidopsis*, rice and soybean. A total of 139 *NACs* from soybean, 78 *NACs* from *Arabidopsis* and 32 *NACs* from rice were used to construct the NJ tree with 1000 bootstrap based on the full length protein sequences of *NACs*. The NAC proteins are grouped into 17 distinct groups

Phylogenetic relationships exists between *NAC* family proteins in *Arabidopsis*, rice and soybean and for clarification of these complex relationships, an unrooted tree can be constructed using NAC protein sequences from rice, *Arabidopsis* and soybean. Most of the groups obtained from the results were similar to previous phylogenetic analyses [42, 43]. All the NAC members were clustered in the phylogenetic tree into 17 groups and this is shown in (Fig. 1).

*GmNAC* geness are as diverse as NAC proteins and in 16 groups there was unequal distribution of 139 *GmNACs* was found. In Group IV no member of *GmNACs* was present. In the phylogenetic analysis of *Arabidopsis* and rice this similar formula was observed [18, 42], which indicates that either this group is acquired in rice and *Arabidopsis* or is lost in soybean plant after their common ancestors went through the process of divergence. Move over in rice and *Arabidopsis* plants, certain specific roles are played by these *NACs* and for studying the relationship between different plant species understanding of characteristics of these groups is important.

Usually similar functions are performed by the genes which have a close evolutionary relationship. Predicting the functions of gene is important in many functional studies and polygenetic analysis can be used for this purpose. Furthermore study of phylogenetic relationships is effective for prediction of stress-related functional genes [24, 42]. From the phylogenetic tree, it can be established that *GmNAC100*, *GmNAC101, GmNAC102, GmNAC103 GmNAC118* and *GmNAC119* might play important part in the formation and development of shoot apical meristem as these are combined together in single with *CUC1* and *CUC3* [44, 45]. *OsNAC52*, *ANAC017, ONAC063, GmNAC004, GmNAC005, GmNAC116* and *GmNAC117* which are combined together in a single group are responsible for drought tolerance and oxidative stress in transgenic plants [36, 37, 46].

A close evolutionary relationship is shown by *GmNAC020* and *GmNAC021* with *ANAC096*. ABF2 and ABF4 functions synergistically with *ANAC096* and through their combined function plants can survive under osmotic stress and dehydration [47]. *GmNAC064* to *GmNAC082* are orthologous with *ORE1/ANAC092/AtNAC2, NAC047, OsNAC1, ANAC072/RD26, ANAC019, OsNAC9, OsNAC6, ANAC032, ANAC055* and *ANAC102* and their functions in plants is to respond to stress conditions thereby resulting in improved yield in different stress conditions such as limited water [30, 34, 48–51]. From *GmNAC128* to *GmNAC139*, *VNDs* (including *VND1-VND7*) and *NSTs* (including *NST1, NST2, NST3/SND1*) combined together in a single group are considered as regulators in vascular vessel development [52]. In Group *XVI, OsNAC10* and *ANAC042* are present and they are responsible for oxidative stress response functions [33, 53]. From *GmNAC008* to *GmNAC009* were orthologues to *VN12* which functions in environmental stress responses and senescence and also interacts with gemeniviral replication initiator protein [54]. Drought-resistance response is inducted by *GmNAC040, GmNAC041, GmNAC042, GmNAC043, NTL6* and *NTL9* which are combined together in a single group and lead senescence is also affected by this drought-resistance response [55, 56]. For cold response and co-ordination of flowering time, an important role is played by *GmNAC046, GmNAC047, GmNAC052, GmNAC053* and *LOV1* which are clustered in one group [57]. These results indicated different possible functions which are performed by *NAC* genes in the soybean plant.

### Structural and motifs analysis of *GmNAC* genes

**Fig. 2:**
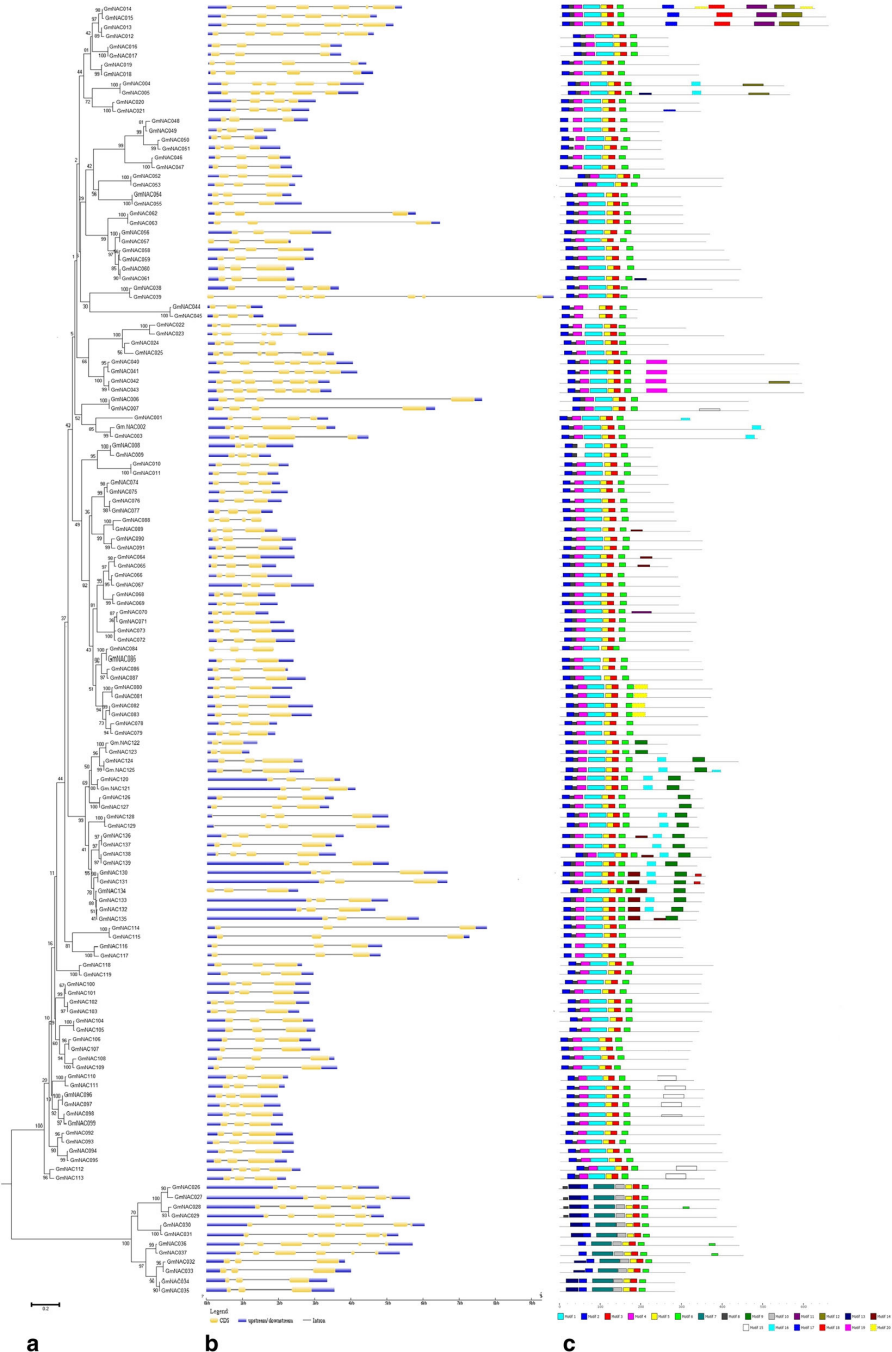
Phylogenetic relationships, gene structure and motif compositions of soybean *NAC* genes. **a** Multiple alignments of 139 full length proteins of *NAC* genes from Soybean were executed by MEGA 6.0 to construct the phylogenetic tree by the Neighbour-Joining (NJ) method with 1,000 bootstrap replicates. MEGA6.0 by the Neighbor-Joining (NJ) method with 1,000 bootstrap replicates. **b** Exon/intron structures of *NAC* genes from Soybean. Exons and introns are represented by blue, yellow boxes and black lines, respectively. **c** Schematic representation of the conserved motifs in the NAC proteins from Soybean elucidated by MEME. Each motif is represented by a colored box. The black lines represent the non-conserved sequences. Refer to Additional file 3 for the details of individual motif

During the evolution period of multigene families, the structure of gene was commonly diversified and this facilitates the evolutionary co-option of genes for adopting different new functions in accordance with changes in the environment [21]. For in-depth investigation of structure of soybean *NAC* genes, we analyzed conserved motifs and intron/exon distribution according to the phylogenetic relationships (Fig. 2).

The analysis of gene structure indicates that introns present in *GmNACs* genes varies from 1 to 7 and this is similar to banana, in which the number of introns vary from 0 to 6. [58]. Nevertheless, the introns in cotton and rice vary from 0 to 9 and 0 to 16 respectively [21, 59]. Through these results it is suggested that there exist small diversity in the gene structure of *GmNACs* genes when compared to *NAC* genes present in cotton and rice. It was also found that in 94 of 139 *GmNAC* genes two introns were present. In cotton, rice and banana this phenomena was observed and two introns were present in majority of *NAC* genes [21, 58, 59]. Nuruzzaman et al. [21] suggested that in rice the intron loss is faster compared to intron gain after the process of segmental duplication. Furthermore, 7 introns are present in *GmNAC039*, and exon-intron structure is similar in most of the *GmNAC* members which are present in the same group. In each group and close evolutionary relationship are supported by the conserved intron numbers.

For further examination of diverse structure of soybean NAC proteins, MEME program along with subsequent annotation with InterPro was used and 20 conserved motifs were identified and predicted (Fig. 2). In all the *GmNACs* there were at least 5 of the 7 main motifs present (motif 1, 2, 4, 5, 6, 7, 8 and 10). These motifs were annotated as no apical meristem (NAM) or as NAC domain, and therefore all soybean *NACs* which are identified in the study possessed conserved features which are present in the *NAC* family. The conserved motifs which are present in the N-region of the NAC proteins provides functions for DNA-binding and this indicated that these motifs are very important for proper functioning of NAC proteins and Singh et al. [60] observed a similar phenomenon for *NACs* present in potato. The motif number 19 was particularly found in 4 genes only. Moreover Motif 1 was similar to one present in *CUC3* gene which is involved in leaf development and abiotic stress responses [45]. In the *AtSOGI* motifs 7 and 10 were found which provides response to DNA damage [61]. In the *VNDs* of *Arabidopsis NAC* genes Motif 9 and 16 were found and these were also associated with regulation of xylem vessel for example *AtVND4, AtVND5, AtVND2, AtVND6* and also performed functions in biosynthesis of vessels [52]. Similar motif composition is shared by NAC proteins which are combined together in a same group and this indicates that members present is same group possess similar functionalities.

### Differential expression profiles of the 139 *GmNAC* genes using previous results of previous studies

The productivity of soybean is severely affected by drought stress and abiotic stress and therefore study of genes of this crop important to increase crop yield. The research conducted by Dung et al. (2011) [23] indicated that among 152 *GmNACs* having putative full-length open reading frame, about fifty stress-related genes have been predicted using phylogenetic analyses of *ANAC, GmNAC* and *ONAC* families. According to the results of their study seven of our 139 *GmNAC* genes (namely *GmNAC033, 064, 065, 068, 066, 067and 069*) were found ubiquitously in all eight tissues, while 22 genes were found expressed strongly in stressed leaves as shown (Additional file 4). Silico analysis of *GmNACs* was conducted in the study along with rice and *Arabidopsis* counterparts and similar *NAC* architecture was revealed [23]. These results indicate that the stress-related *NAC* genes are differentially expressed spatially and temporally in response to abiotic stresses.

**Fig. 3:**
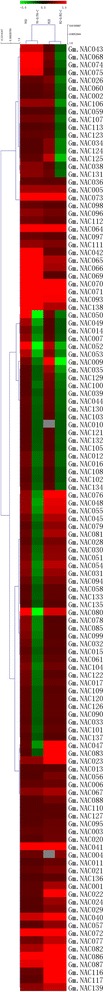
Differential expression data of 139 *GmNAC* genes in V6 and R2 leaves under drought stress. Genes shown are either up-regulated or down-regulated at least by two-fold; the colors indicate expression intensity (*red*, high expression; *green*, low expression; *grey*, no expression). 66 K Affymetrix Soybean Array GeneChip source: Dung Tien Le et al. [41] see Additional file 7

Later on, the study of Dung Tien Le et al. [41] indicated that our 139 *GmNAC* genes displayed different expression profiles in soybean leaves at V6 and R2 stages under drought stress as shown in (Fig. 3). Among these genes, *GmNAC005, GmNAC040, GmNAC041*, *GmNAC064, GmNAC065, GmNAC070, GmNAC071, GmNAC072, GmNAC073, GmNAC086, GmNAC087* and *GmNAC093* genes were upregulated at both V6 and R2 stages with at least two-fold change (drought vs. well-water). Interestingly, Nguyen Phuong Thao, et al. [62] suggested that among our 139 *GmNAC* genes, *GmNAC064*, *GmNAC065*, *GmNAC070*, *GmNAC071*, *GmNAC072* and *GmNAC111* genes were not only drought-inducible but also highly upregulated in soybean roots of drought-tolerant cultivar with at least two-fold change (unstressed vs. stressed conditions).

In the above discussion, different biological aspects of *GmNAC* TFs have been identified and it can be considered that there is a need of future follow-up studies for improving the understanding of different regulatory functions performed by *NAC* members. If the operations of TFs are made understandable to greater extends then this understanding can be translated into potential application for increasing plant productivity.

### Predicted *GmNAC* genes and dehydration stress: analysis of expression patterns among drought-tolerant and drought-sensitive soybean cultivars

As previously discussed, dehydration stress has altered expression of many *GmNAC* genes in the soybean leaves [23, 41]. Pinpointing the specific function of genes is of great importance in agriculture and other related industries. It is, after all, through such means that superior cultivars could be developed with great accuracy. In this study, the goal was to gather information on gene expression patterns in relation to dehydration stress, potentially revealing genes that could allow for new drought-tolerant varieties to be engineered. The study involved the analysis of 28 predicted stress-related *GmNAC* genes, mainly done through qRT-PCR. As to be expected, gene-specific primers were utilized (Additional file 5), and the samples were subjected to simulated drought conditions.

**Fig. 4:**
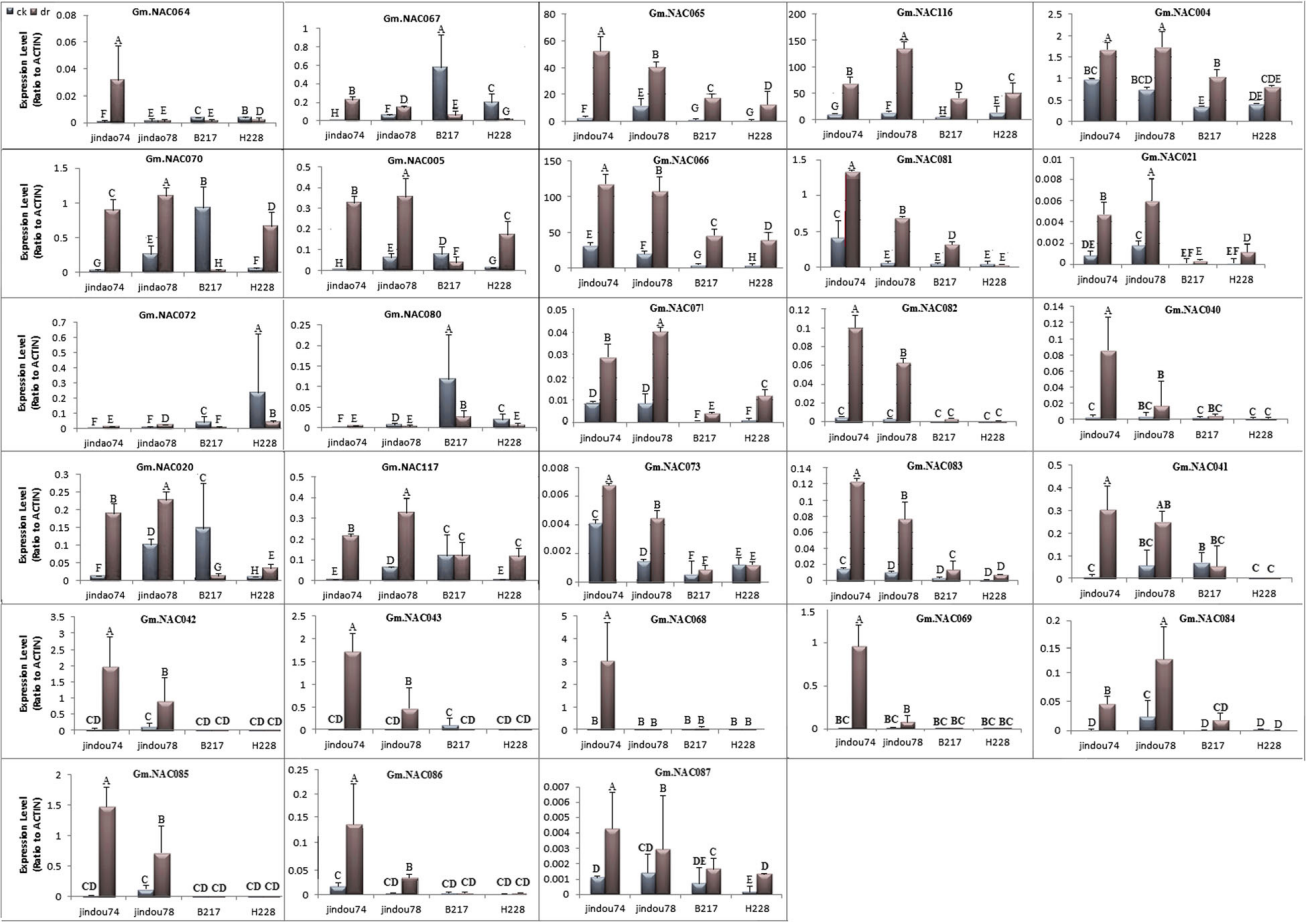
Expression of 28 selected dehydration-inducible *GmNAC* genes in leaf tissues of soybean plants. qPCR analysis of the selected genes of drought sensitive (B217, H228) and drought tolerant (Jindou74 and 78) cultivars under normal (ck.) and drought (dr.) conditions. Error bars above means denote LSD value of three replicates. Different alphabetical letters (a, b, c…) above means show the significant differences (LSD < 0.05) among treatments of a cultivar at control and 8 days after drought. Error bars above means denote LSD value of three biological replicates

Despite being subjected to the same conditions, though as to be expected, the drought-tolerant and drought-sensitive cultivars had different phenotypic expressions in response to drought. Both H288 and B217 (drought-sensitive varieties) exhibited wilting at a much greater extent compared to the Jindou 74 and 78 (drought-tolerant varieties). The impact on growth was also much more prominent on the former pair than on the latter. These effects became more noticeable as the experiment progressed, and the differences were most evident upon reaching the eighth day (Additional file 6). As for gene expression, the *GmNAC* genes analyzed were expressed differently among drought-tolerant and drought-sensitive cultivars (Fig. 4). The drought-tolerant varieties (which include Jindou 74 and 78) exhibited high expression levels for *GmNAC004, GmNAC021, GmNAC065, GmNAC066, GmNAC073, GmNAC082, GmNAC083* and *GmNAC087*. The drought-sensitive cultivars (B217, H228), on the other hand, were detected to have lower expressions for the aforesaid genes. Do note that this trend was observed in both extreme drought and standard stress setups. Interestingly, the Jindou varieties still maintained higher expressions for all genes mentioned so far, even under non-stress conditions. Although associated with higher expression in more genes while under drought stress (24 out of 28 *GmNAC* genes to be exact), the Jindou varieties failed to match H228 and B217’s expression for *GmNAC067, GmNAC072*, and *GmNAC080* under normal conditions (Fig. 4).

Furthermore, the drought-linked *GmNAC* genes (*GmNAC004, GmNAC021, GmNAC065, GmNAC066, GmNAC073, GmNAC082, GmNAC083* and *GmNAC087*) were found to be inducible regardless of drought sensitivity of cultivars, although in both drought and non-drought conditions, such genes showed greater transcript levels in the Jindou or drought-tolerant soybean varieties than that in drought-sensitive varieties. The same set of genes were also upregulated throughout different stages of leaf growth. The results suggest that these genes may play conserved roles in leaf growth and in plant responses to drought stresses.

## Conclusion

Based on the data gathered throughout the course of the study, it may be said that drought tolerance is influenced by the expression of certain *GmNAC* genes, particularly *GmNAC004, GmNAC021, GmNAC065, GmNAC066, GmNAC073, GmNAC082, GmNAC083* and *GmNAC087*. It is crucial to mention, however, that only three among the six have been previously known to be connected to drought. It may also be concluded that there is a correlation between gene expression, transcriptional regulation, and superior tolerance to drought, as seen from the different stages of leaf development across tolerant and sensitive cultivars. All in all, the study has revealed a number of potential gene candidates that could be given focus by those attempting to develop soybean cultivars with even greater drought tolerance.

## Methods

### Identification of soybean *NAC* genes

Putative NAC proteins in *G. Max* were identified by a BLAST search at Phytozome v 10.0 [63] using well known NAC proteins from *Arabidopsis* and rice as guidance for the criterion of the gene without *NAC*. The NAM domains were expelled from the analysis. The database of *Arabidopsis* and rice were extracted from the National Centre for Biotechnology Information [64]. The search was based upon NAC transcription factor as a key word. Approximately 400 protein sequences were carried out. Upon removal of the putative, redundant, and unknown sequences, a total of 110 *NACs* from *Arabidopsis* and rice remained. All candidate *GmNACs* that contained at least one NAC domain (PF02365) were confirmed using SMART [65] and Pfam [66] online software. Lastly, 139 non-redundant and complete NAC-domain containing protein sequences were selected for further analysis. The genes and corresponding Gene bank accession numbers for *Arabidopsis* and rice are listed in Additional file 2.

### Phylogenetic analysis

The finalized 139 *G. Max* protein sequences that contained NAC-domain, 78 *Arabidopsis* NAC proteins, and 32 rice NAC proteins sequences in FASTA format were aligned for the construction of an un-rooted phylogenetic tree by the neighbor-joining method. The confidence level of the monophyletic groups was estimated using a bootstrap analysis of 1000 replicates. A multiple sequence alignment (MSA) and phylogenetic analysis were performed using quicktree [67]. Finally, the evolutionary tree is designed by MEGA6 or figtree. The NAC protein sequences of the phylogenetic tree are listed in Additional file 8.

We utilized the Gene structure display server program GSDS [68] in order to show the exon/intron structure of each *GmNAC* gene by comparison of the coding sequences with their corresponding genomic sequences from Phytozome.

**A MEME 4.10.1** program utility [69] was used for the display of motifs and sequence logos of the *GmNAC* proteins from soybean [70]. The parameters were set as:

- Motif Count: Search for 20 motifs
- Occurrence of a single motif: 0 to 1 occurrence per sequence of a contributing motif site
- Motifs width range: between 6 and 50 wide - inclusive

All motifs discovered by MEME were annotated by adopting Interpro [71] and SMART [64]. The transmembrane domain into the NAC proteins was expected via the ARAMEMNON plant membrane protein database [72]. Motif information was listed in Additional file 3.

### Plant materials, growth conditions and treatments

Four soybean varieties viz., (Jindou74 and 78) drought tolerant and (H228 and B217) drought sensitive were sown in each pot on 10 May 2015. Most of the seedlings emerged within 7 days after sowing. Plants of each pot received adequate watering regularly to maintain optimal soil moisture until the water stress treatment was imposed. Plants of all the genotype were subjected to two levels of water regime viz., ck: Non-stress (Control); water was applied when it is required and dr: Drought stress throughout the growing period; while the plant has two *trifolium* leaves, they were affected to drought stress by holding water. For Quantitative real-time RT-PCR, leaves of v2 growth phase were collected from control plant and plant with eight days of drought treatment as appearance of wilting symptom was clearly observed comparing with the control plant, and the results are shown at Additional file 6.

### RNA isolation and cDNA synthesis

Pre-frozen leaves of control and stress samples were ground to a fine powder in liquid nitrogen; and total RNA was isolated using the TRIzol reagent (Invitrogen) according to the manufacturer’s instructions. The obtained RNA concentration and purity were measured using a spectrophotometer (NanoDrop, ND-1000), and contaminating DNA in the total RNA was degraded using RNase-free DNase, according to the manufacturer’s instructions. High-quality 2 μg RNA was utilized to obtain the first strand of cDNAs from each sample synthesis kit (M-MLV, China), according to the manufacturer’s protocol. The quality of the cDNA and contamination with genomic DNA were examined using (NanoDrop, ND-1000) and a standard PCR assay with primers of the *β*-actin soybean gene.

### Quantitative real-time PCR and statistical analyses

cDNA was diluted 1:10 with ddH_2_O for (qRT-PCR) assay, Gene-specific primers were designed for the 28 *GmNACs* genes, according to their predicted CDS and gene structure using PrimerQuest IDT (http://www.idtdna.com/primerquet/home/index/). Quantitative real-time RT-PCR (qPCR) was performed on a qPCRsoft V 3.0. Using NI – ROX DYE I according to the manufacturer’s protocol the reaction mixture contained 10 μL of SYBR Green I Master Mix (Applied Biosystems), 0.8 μm of each primer and 2 μL diluted template cDNA. The qPCR assays were performed with three replicates. The amplification programs the following cycling condition: 95 °C for 3 min, 45 cycles of 95 °C for 10 s, 58 °C for 30 s, and 72 °C for 30 s. each sample had three replicates to ensure the accuracy of results, and reaction mixtures with β -actin of glyma templates were used as controls to evaluate the specificity of each real-time PCR. The relative gene expression levels were determined by the ΔΔCT method as described previously [73]. The genes ID with specific primers information were listed in Additional file 5.

### Statistical analysis

The experiment was arranged in a completely randomized design (CRD) with three independent replicates. Each replicate was consisted of four pots. Analysis of Variance (ANOVA) was used for analyzing the data statistically. For the significant difference, a margin of 5% probability level was used utilizing Statistix 8.1 (Analytical Software, Tallahassee, FL, USA) software.

## Additional files

Additional file 1: Table of *GmNACs* data. (DOCX 23 kb)
Additional file 2: Table of gene’s names and gene bank accession number of *Arabidopsis* and Rice. (DOCX 87 kb)
Additional file 3: Table of motifs information. (DOCX 14 kb)
Additional file 4: Heat map representation for tissue-specific expression of 44 predicted stress-responsive and 6 previously reported dehydration-responsive *GmNAC* genes. Expression patterns of 44 *GmNAC* genes were analysed using Illumina transcriptome data (source: [22]). Box A indicates a group of ubiquitously expressed *GmNAC* genes in the eight types of tissues examined. The colors indicate expression intensity (red, high expression; green, low expression; grey, no expression). (JPG 74 kb)
Additional file 5: Table of specific primers information. (DOCX 21 kb)
Additional file 6 Phenotypes of drought at different times of drought treatments for four selected varieties: drought tolerant (Jindou 74 and 78) and sensitive (H228, B217) varieties in (a) and (b) as follow: (a) whole plants of: control, 4 days of drought treatments, and 8 days of drought treatments. (b) The leaves of control, 4 days, 8 days of drought treatments. (JPG 265 kb)
Additional file 7: The data of 139 *GmNACs* of Fig. 3. (source: 66 K Affymetrix Soybean Array GeneChip of Dung Tien Le et al. [41]. (DOCX 22 kb)
Additional file 8: NACs protein sequences of phylogenetic tree. (TXT 91 kb)

## Abbreviations

*ANAC, AtNAC*: *Arabidopsis NAC*
cDNA: Denotes complementary DNA. cDNA is synthesized or manufactured from an mRNA (messenger RNA template)
CDS: Coding DNA sequence that portion of a gene’s DNA or RNA, composed of exons, that code for protein
*Gm*: Glycine Max
HSFs: Heat stress transcription factors
*NAC*: *NAM, ATAF1/2* and *CUC* transcription factors
*NST*: *NAC* secondary wall thickening promoting factor
*ONAC, OsNAC*: *Oryza sativ NAC*
QRT-PCR: Quantitative real-time PCR
*SND*: Secondary wall-associated *NAC* domain protein
TFs: Transcription factors
TR: Transcriptional regulatory
*VND*: Vascular-related NAC- domain

## References

[1] Food and Agriculture Organization of the United Nations, FAOSTAT. Rome, Italy. FAO (2009). Available at: http://faostat.fao.org (Accessed 3 Dec 2009).

[2] Soybean Production (Food and Agriculture Organization of the United Nations—FAO) [(Accessed on 15 Oct 2013)]. Available online: http://faostat3.fao.org/faostat-gateway/go/to/download/Q/QC/E.

[3] Tran LS, Quach TN, Guttikonda SK, Aldrich DL, Kumar R, Neelakandan A, Valliyodan B, Nguyen HT (2009). Molecular characterization of stress-inducible *GmNAC* genes in soybean. Mol Genet Genomics.

[4] United States Department of Agriculture (USDA). World soybean production 2016/2017. https://www.worldsoybeanproduction.com/.

[5] Mohammad M (2016). Abiotic and biotic stresses in soybean production, soybean production: Book, Volume 1.

[6] Dai A (2013). Increasing drought under global warming in observations and models. Nat Climate Change.

[7] Foyer C H, Lam HM., Nguyen HT, Siddique KHM, Varshney R, et al. Neglecting legumes has compromised global food and nutritional security. Nat Plants 2016 (in press). 10.1038/nplants.2016.112 28221372

[8] Ku Y-S, Au-Yeung W-K, Yung Y-L, Li M-W, Wen C-Q, Liu X, Board JE (2013). Drought stress and tolerance in soybean. A comprehensive survey of international soybean research - genetics, physiology, agronomy and nitrogen relationships.

[9] Sablowski RWM, Meyerowitz EM (1998). A homolog of *NO APICAL MERISTEM* is an immediate target of the floral homeotic genes *APETALA3*/*PISTILLATA*. Cell.

[10] Souer E, van Houwelingen A, Kloos D, Mol J, Koes R (1996). The No Apical Meristem gene of petunia is required for pattern formation in embryos and flowers and is expressed at meristem and primordia boundaries. Cell.

[11] Jensen MK, Hagedorn PH, de Torres-Zabala M, Grant MR, Rung JH, Collinge DB (2008). Transcriptional regulation by a NAC (NAM-ATAF1,2-CUC2) transcription factor attenuates ABA signalling for efficient basal defence towards *Blumeria graminis* f. sp. *hordei* in *Arabidopsis*. Plant J.

[12] Aida M, Ishida T, Fukaki H, Fujisawa H, Tasaka M (1997). Genes involved in organ separation in *Arabidopsis*: an analysis of the cup-shaped cotyledon mutant. Plant Cell.

[13] Delessert C, Kazan K, Wilson IW, Van Der Straeten D, Manners J, Dennis ES, Dolferus R (2005). The transcription factor ATAF2 represses the expression of pathogenesis-related genes in *Arabidopsis*. Plant J.

[14] Lu PL, Chen NZ, An R, Su Z, Qi BS, Ren F, Chen J, Wang XC (2007). A novel drought-inducible gene, ATAF1, encodes a NAC family protein that negatively regulates the expression of stress-responsive genes in *Arabidopsis*. Plant Mol Biol.

[15] Wang X, Basnayake BM, Zhang H, Li G, Li W, Virk N, Mengiste T, Song F (2009). The *Arabidopsis* ATAF1, a NAC transcription factor, is a negative regulator of defense responses against necrotrophic fungal and bacterial pathogens. Mol Plant Microbe Interact.

[16] Kikuchi K, Ueguchi-Tanaka M, Yoshida KT, Nagato Y, Matsusoka M, Hirano HY (2000). Molecular analysis of the *NAC* gene family in rice. Mol Gen Genet.

[17] Duval M, Hsieh TF, Kim SY, Thomas TL (2002). Molecular characterization of *AtNAM*: a member of the *Arabidopsis* NAC domain superfamily. Plant Mol Biol.

[18] Ooka H, Satoh K, Doi K, Nagata T, Otomo Y, Murakami K (2003). Comprehensive analysis of *NAC* family genes in *Oryza sativa* and *Arabidopsis thaliana*. DNA Res.

[19] Wang N, Zheng Y, Xin H, Fang L, Li S (2013). Comprehensive analysis of NAC domain transcription factor gene family in *Vitis vinifera*. Plant Cell Rep.

[20] Satheesh V, Jagannadham PTK, Chidambaranathan P, Jain PK, Srinivasan R (2014). NAC transcription factor genes: genome-wide identification, phylogenetic, motif and *cis*-regulatory element analysis in pigeonpea (*Cajanus cajan* (L.) Millsp.). Mol Biol Rep.

[21] Nuruzzaman M, Manimekalai R, Sharoni AM, Satoh K, Kondoh H, Ooka H (2010). Genome-wide analysis of NAC transcription factor family in rice. Gene.

[22] Puranik S, Sahu PP, Mandal SN BVS, Parida SK, Prasad M (2013). Comprehensive genome-wide survey, genomic constitution and expression profiling of the NAC transcription factor family in foxtail millet (*Setaria italica* L.). PLoS One.

[23] Le DT, Nishiyama R, Watanabe Y, Mochida K, Yamaguchi-Shinozaki K, Tran LS (2011). Genome-wide survey and expression analysis of the plant-specific NAC transcription factor family in soybean during development and dehydration stress. DNA Res.

[24] Liu TK, Song XM, Duan WK, Huang ZN, Liu GF, Li Y (2014). Genome-wide analysis and expression patterns of NAC transcription factor family under different developmental stages and abiotic stresses in Chinese cabbage. Plant Mol Biol Rep.

[25] Yamaguchi M, Ohtani M, Mitsuda N, Kubo M, Ohme-Takagi M, Fukuda H (2010). *VND-INTERACTING2*, a NAC domain transcription factor, negatively regulates xylem vessel formation in *Arabidopsis*. Plant Cell.

[26] Guo YF, Gan SS (2006). *AtNAP*, a NAC family transcription factor, has an important role in leaf senescence. Plant J.

[27] Hao YJ, Wei W, Song QX, Chen HW, Zhang YQ, Wang F (2011). Soybean NAC transcription factors promote abiotic stress tolerance and lateral root formation in transgenic plants. Plant J.

[28] Shan W, Kuang JF, Chen L, Xie H, Peng HH, Xiao YY (2012). Molecular characterization of banana NAC transcription factors and their interactions with ethylene signalling component EIL during fruit ripening. J Exp Bot.

[29] Nambara E, Marion-Poll A (2005). Abscisic acid biosynthesis and catabolism. Annu Rev Plant Biol.

[30] Bu QY, Jiang HL, Li CB, Zhai QZ, Zhang JY, Wu X (2008). Role of the *Arabidopsis thaliana* NAC transcription factors *ANAC019* and *ANAC055* in regulating *jasmonic* acid-signaled defense responses. Cell Res.

[31] Tran LS, Nakashima K, Sakuma Y, Simpson SD, Fujita Y, Maruyama K (2004). Isolation and functional analysis of *Arabidopsis* stress inducible NAC transcription factors that bind to a drought responsive cis-element in the early responsive to dehydration stress 1 promoter. Plant Cell.

[32] Hu H, Dai M, Yao J, Xiao B, Li X, Zhang Q (2006). Overexpressing a *NAM*, *ATAF*, and *CUC* (NAC) transcription factor enhances drought resistance and salt tolerance in rice. Proc Natl Acad Sci U S A.

[33] Jeong JS, Kim YS, Baek KH, Jung H, Ha SH, Do Choi Y (2010). Root-specific expression of *OsNAC10* improves drought tolerance and grain yield in rice under field drought conditions. Plant Physiol.

[34] Nakashima K, Tran LS, Van Nguyen D, Fujita M, Maruyama K, Todaka D, Ito Y, Hayashi N, Shinozaki K, Yamaguchi-Shinozaki K (2007). Functional analysis of a NAC-type transcription factor *OsNAC6* involved in abiotic and biotic stress-responsive gene expression in rice. Plant J.

[35] Hu H, You J, Fang Y, Zhu X, Qi Z, Xiong L (2008). Characterization of transcription factor gene *SNAC2* conferring cold and salt tolerance in rice. Plant Mol Biol.

[36] Yokotani N, Ichikawa T, Kondou Y, Matsui M, Hirochika H, Iwabuchi M, Oda K (2009). Tolerance to various environmental stresses conferred by the salt-responsive rice gene *ONAC063* in transgenic *Arabidopsis*. Planta.

[37] Zheng X, Chen B, Lu G, Han B (2009). Overexpression of a *NAC* transcription factor enhances rice drought and salt tolerance. Biochem Bioph Res Co.

[38] Meng Q, Zhang C, Gai J, Yu D (2007). Molecular cloning, sequence characterization and tissue-specific expression of six *NAC*-like genes in soybean (*Glycine max* (L.) Merr.). J Plant Physiol.

[39] Tran LSP, Quach TN, Guttikonda SK, Aldrich DL, Kumar R, Neelakandan A, Valliyodan B (2009). Nguyen HT Molecular characterization of stress-inducible *GmNAC* genes in soybean. Mol Genet Genomics.

[40] Pinheiro GL, Marques CS, Costa MDBL, Reis PAB, Alves MS, Carvalho CM, Fietto LG, Fontes EPB (2009). Complete inventory of soybean NAC transcription factors: Sequence conservation and expression analysis uncover their distinct roles in stress response. Gene.

[41] Le DT, Nishiyama R, Watanabe Y, Tanaka M, Seki M, Yamaguchi-Shinozaki K, Shinozaki K, Tran L-SP (2012). Differential gene expression in soybean leaf tissues at late developmental stages under drought stress revealed by genome-wide transcriptome analysis. PLoS One.

[42] Hu W, Wei Y, Xia Z, Yan Y, Hou X, Zou M (2015). Genome-wide identification and expression analysis of the NAC transcription factor family in *Cassava*. PLoS One.

[43] Balazadeh S, Kwasniewski M, Caldana C, Mehrnia M, Zanor MI, Xue GP (2011). ORS1, an H(2)O(2)-responsive NAC transcription factor, controls senescence in *Arabidopsis thaliana*. Mol Plant.

[44] Hibara K, Takada S, Tasaka M (2003). *CUC1* gene activates the expression of *SAM*-related genes to induce adventitious shoot formation. Plant J.

[45] Hibara K, Karim MR, Takada S, Taoka K, Furutani M, Aida M, Tasaka M (2006). *Arabidopsis CUP-SHAPED COTYLEDON3* regulates postembryonic shoot meristem and organ boundary formation. Plant Cell.

[46] Ng S, Ivanova A, Duncan O (2013). A membrane-bound *NAC* transcription factor, *ANAC017*, mediates mitochondrial retrograde signaling in *Arabidopsis*. Plant Cell.

[47] Xu ZY, Kim SY, Hyeon do Y, Kim DH, Dong T, Park Y, Jin JB, Joo SH, Kim SK, Hong JC, Hwang D, Hwang I (2013). The *Arabidopsis* NAC transcription factor *ANAC096* cooperates with *bZIP-type* transcription factors in dehydration and osmotic stress responses. Plant Cell.

[48] Patil M, Ramu SV, Jathish P (2014). Overexpression of *AtNAC2* (*ANAC092*) in groundnut (*Arachis hypogaea* L.) improves abiotic stress tolerance. Plant Biotechnol Rep.

[49] Balazadeh S, Siddiqui H, Allu AD, Matallana-Ramirez LP, Caldana C, Mehrnia M (2010). A gene regulatory network controlled by the NAC transcription factor *ANAC092/AtNAC2/ORE1* during salt- promoted senescence. Plant J.

[50] Mahmood K, El-Kereamy A, Kim SH, Nambara E, Rothstein SJ (2016). *ANAC032* positively regulates age-dependent and stress-induced senescence in Arabidopsis *thaliana*. Plant Cell Physiol.

[51] Fujita M, Fujita Y, Maruyama K, Seki M, Hiratsu K, Ohme-Takagi M, Tran LS, Yamaguchi-Shinozaki K, Shinozaki K (2004). A dehydration-induced NAC protein, *RD26*, is involved in a novel ABA-dependent stress-signaling pathway. Plant J.

[52] Zhong R, Lee C, Ye ZH (2010). Evolutionary conservation of the transcriptional network regulating secondary cell wall biosynthesis. Trends Plant Sci.

[53] Wu A, Allu AD, Garapati P, Siddiqui H, Dortay H, Zanor MI (2012). *JUNGBRUNNEN1*, a reactive oxygen species-responsive NAC transcription factor, regulates longevity in *Arabidopsis*. Plant Cell.

[54] Yang SD, Seo PJ, Yoon HK (2011). The *Arabidopsis* NAC transcription factor *VNI2* integrates abscisic acid signals into leaf senescence via the *COR/RD* genesThe *Arabidopsis* NAC transcription factor *VNI2* integrates abscisic acid signals into leaf senescence via the *COR/RD* genes. Plant Cell.

[55] Kim MJ, Park MJ, Seo PJ, Song JS, Kim HJ, Park CM (2012). Controlled nuclear import of the transcription factor *NTL6* reveals a cytoplasmic role of *SnRK2.8* in the drought-stress response. Biochem J.

[56] Yoon HK, Kim SG, Kim SY, Park CM (2008). Regulation of leaf senescence by *NTL9*-mediated osmotic stress signaling in *Arabidopsis*. Mol Cells.

[57] So Yeon Y, Yunhee K, Soo Young K, Jong Seob L, Ji Hoon A (2007). Control of flowering time and cold response by a NAC-Domain protein in *Arabidopsis*. PLoS One.

[58] Cenci A, Guignon V, Roux N, Rouard M (2014). Genomic analysis of NAC transcription factors in banana (*Musa acuminata*) and definition of *NAC* orthologous groups for *monocots* and *dicots*. Plant Mol Biol.

[59] Shang H, Li W, Zou C, Yuan Y (2013). Analyses of the *NAC* transcription factor gene family in *Gossypium raimondii* Ulbr. chromosomal location, structure, phylogeny, and expression patterns. J Integr Plant Biol.

[60] Singh AK, Sharma V, Pal AK, Acharya V, Ahuja PS (2013). Genome-wide organization and expression profiling of the NAC transcription factor family in potato (*Solanum tuberosum* L.). DNA Res.

[61] Yoshiyama KO, Kimura S, Maki H, Britt AB, Umeda M. The role of *SOG1*, a plant-specific transcriptional regulator, in the DNA damage response. Plant Signal Behav. 2014;9:e28889. doi:10.4161/psb.28889. 10.4161/psb.28889 PMC4091597 24736489

[62] Thao PH, Thu NBA, Hoang XL, Ha CV, Tran LS (2013). Differential expression analysis of a subset of drought-responsive *GmNAC* genes in Two soybean cultivars differing in drought tolerance. Int J Mol Sci.

[63] Phytozome quick search. 2016. Retrieved from http://phytozome.jgi.doe.gov/.

[64] National Centre for Biotechnology Information Search Engine. 2016. Retrieved from http://www.ncbi.nlm.nih.gov/.

[65] Letunic I, Copley RR, Schmidt S, Ciccarelli FD, Doerks T, Schultz J, Ponting CP, Bork P (2004). SMART 4.0: towards genomic data integration. Nucleic Acids Res.

[66] Finn RD, Mistry J, Schuster-Bockler B, Griffiths-Jones S, Hollich V, Lassmann T, Moxon S, Marshall M, Khanna A, Durbin R, Eddy SR, Sonnhammer EL, Bateman A (2006). Pfam: clans, web tools and services. Nucleic Acids Res.

[67] Mailund T, Pedersen C (2004). QuickJoin--fast neighbour-joining tree reconstruction. Bioinformatics.

[68] Guo AY, Zhu QH, Chen X, Luo JC (2007). [GSDS: a gene structure display server]. Yi Chuan.

[69] MEME Suite 4.10.1 Patches. 2016. Retrieved from http://meme-suite.org/meme-software/4.10.1/readme.html.

[70] Bailey T, Boden M, BuskeF A, Frith M (2009). MEME suite: tools for motif discovery and searching. Nucleic Acids Res.

[71] Interpro. 2016. Retrieved from http://www.ebi.ac.uk/interpro/.

[72] ARAMEMNON. 2016. Retrieved from http://aramemnon.botanik.uni-koeln.de/

[73] Livak KJ, Schmittgen TD (2001). Analysis of relative gene expression data usingreal-time quantitative PCR and the 2(−Delta Delta C(T)) Method. Methods.

