# Supplementary material for: The essence of NAC gene family to the cultivation of drought-resistant soybean (Glycine max L. Merr.) cultivars
Source: BMC Plant Biol. 2017 Feb 28;17:55. doi: 10.1186/s12870-017-1001-y (PMC5330122; doi:10.1186/s12870-017-1001-y)
Supplement: Additional file 1: — Table of GmNACs data. (DOCX 23 kb) [file 12870_2017_1001_MOESM1_ESM.docx]

| *Gene name depending on phylogenetic tree* | Gene_ ID | Gene_ ID another name | |
| --- | --- | --- | --- |
| *Gm.NAC001* | Glyma.08G156500 | GLYMA08G16630 | |
| *Gm.NAC002* | Glyma.06G152900 | GLYMA06G15840 | |
| *Gm.NAC003* | Glyma.04G213300 | GLYMA04G39140 | |
| *Gm.NAC004* | Glyma.20G172100 | GLYMA20G31210 | |
| *Gm.NAC005* | Glyma.10G219600 | GLYMA10G36360 | |
| *Gm.NAC006* | Glyma.20G192500 | GLYMA20G33430 | |
| *Gm.NAC007* | Glyma.10G197500 | GLYMA10G34130 | |
| *Gm.NAC008* | Glyma.18G301500 | GLYMA18G53954 | |
| *Gm.NAC009* | Glyma.08G360200 | GLYMA08G47520 | |
| *Gm.NAC010* | Glyma.08G009700 | GLYMA08G01280 | |
| *Gm.NAC011* | Glyma.05G202300 | GLYMA05G38380 | |
| *Gm.NAC012* | Glyma.18G043900 | GLYMA16G34310 | |
| *Gm.NAC013* | Glyma.11G212400 | GLYMA11G33210 | |
| *Gm.NAC014* | Glyma.14G210000 | GLYMA14G39080 | |
| *Gm.NAC015* | Glyma.02G240500 | GLYMA02G40750 | |
| *Gm.NAC016* | Glyma.16G217400 | GLYMA16G34310 | |
| *Gm.NAC017* | Glyma.09G167400 | GLYMA09G29760 | |
| *Gm.NAC018* | Glyma.15G264100 | GLYMA15G41825 | |
| *Gm.NAC019* | Glyma.08G163100 | GLYMA08G17351 | |
| *Gm.NAC020* | Glyma.17G154100 | GLYMA17G16500 | |
| *Gm.NAC021* | Glyma.05G113000 | GLYMA05G23840 | |
| *Gm.NAC022* | Glyma.07G048100 | GLYMA07G05370 | |
| *Gm.NAC023* | Glyma.16G016700 | GLYMA16G01940 | |
| *Gm.NAC024* | Glyma.16G016400 | GLYMA16G01900 | |
| *Gm.NAC025* | Glyma.07G047900 | GLYMA07G05351 | |
| *Gm.NAC026* | Glyma.19G056400 | GLYMA19G08510 | |
| *Gm.NAC027* | Glyma.16G069300 | GLYMA16G07501 | |
| *Gm.NAC028* | Glyma.19G002900 | GLYMA19G00640 | |
| *Gm.NAC029* | Glyma.05G002700 | GLYMA05G09110 | |
| *Gm.NAC030* | Glyma.02G100200 | GLYMA02G11140 | |
| *Gm.NAC031* | Glyma.01G088200 | GLYMA01G22510 | |
| *Gm.NAC032* | Glyma.13G174700 | GLYMA13G24320 | |
| *Gm.NAC033* | Glyma.07G201800 | GLYMA07G32250 | |
| *Gm.NAC034* | Glyma.12G118700 | GLYMA12G13710 | |
| *Gm.NAC035* | Glyma.06G288500 | GLYMA06G44250 | |
| *Gm.NAC036* | Glyma.08G031900 | GLYMA08G03590 | |
| *Gm.NAC037* | Glyma.05G225100 | GLYMA05G36031 | |
| *Gm.NAC038* | Glyma.13G314600 | GLYMA13G39090 | |
| *Gm.NAC039* | Glyma.12G186900 | GLYMA12G31210 | |
| *Gm.NAC040* | Glyma.14G189300 | GLYMA14G36840 | |
| *Gm.NAC041* | Glyma.02G222300 | GLYMA02G38710 | |
| *Gm.NAC042* | Glyma.06G138100 | GLYMA06G14290 | |
| *Gm.NAC043* | Glyma.04G226700 | GLYMA04G40450 | |
| *Gm.NAC044* | Glyma.15G051200 | GLYMA15G05690 | |
| *Gm.NAC045* | Glyma.08G181100 | GLYMA08G19300 | |
| *Gm.NAC046* | Glyma.19G195800 | GLYMA19G38211 | |
| *Gm.NAC047* | Glyma.03G197900 | GLYMA03G35570 | |
| *Gm.NAC048* | Glyma.13G315300 | GLYMA13G39160 | |
| *Gm.NAC049* | Glyma.12G186200 | GLYMA12G31150 | |
| *Gm.NAC050* | Glyma.12G091200 | GLYMA12G09670 | |
| *Gm.NAC051* | Glyma.11G182000 | GLYMA11G18770 | |
| *Gm.NAC052* | Glyma.17G002800 | GLYMA17G00650 | |
| *Gm.NAC053* | Glyma.07G271100 | GLYMA07G40140 | |
| *Gm.NAC054* | Glyma.12G022700 | GLYMA12G02540 | |
| *Gm.NAC055* | Glyma.11G096600 | GLYMA11G10230 | |
| *Gm.NAC056* | Glyma.19G021900 | GLYMA19G02580 | |
| *Gm.NAC057* | Glyma.13G063300 | GLYMA13G05350 | |
| *Gm.NAC058* | Glyma.18G119300 | GLYMA18G15020 | |
| *Gm.NAC059* | Glyma.08G301100 | GLYMA08G41260 | |
| *Gm.NAC060* | Glyma.02G107000 | GLYMA02G11900 | |
| *Gm.NAC061* | Glyma.01G046800 | GLYMA01G05680 | |
| *Gm.NAC062* | Glyma.18G110700 | GLYMA18G13574 | |
| *Gm.NAC063* | Glyma.08G307100 | | GLYMA08G41995 |
| *Gm.NAC064* | Glyma.14G152700 | | GLYMA14G24220 |
| *Gm.NAC065* | Glyma.13G030900 | | GLYMA02G26480 |
| *Gm.NAC066* | Glyma.06G114000 | | GLYMA06G11970 |
| *Gm.NAC067* | Glyma.04G249000 | | GLYMA04G42800 |
| *Gm.NAC068* | Glyma.06G157400 | | GLYMA06G16440 |
| *Gm.NAC069* | Glyma.04G208300 | | GLYMA04G38560 |
| *Gm.NAC070* | Glyma.13G279900 | | GLYMA13G35550 |
| *Gm.NAC071* | Glyma.12G221500 | | GLYMA12G35000 |
| *Gm.NAC072* | Glyma.12G149100 | | GLYMA12G22880 |
| *Gm.NAC073* | Glyma.06G248900 | | GLYMA06G38410 |
| *Gm.NAC074* | Glyma.20G033300 | | GLYMA20G04400 |
| *Gm.NAC075* | Glyma.07G229100 | | GLYMA07G35630 |
| *Gm.NAC076* | Glyma.02G109800 | | GLYMA02G12220 |
| *Gm.NAC077* | Glyma.01G051300 | | GLYMA01G06150 |
| *Gm.NAC078* | Glyma.15G070300 | | GLYMA15G07620 |
| *Gm.NAC079* | Glyma.13G243200 | | GLYMA13G31660 |
| *Gm.NAC080* | Glyma.13G280000 | | GLYMA13G35560 |
| *Gm.NAC081* | Glyma.12G221400 | | GLYMA12G34990 |
| *Gm.NAC082* | Glyma.12G148900 | | GLYMA12G22790 |
| *Gm.NAC083* | Glyma.06G249100 | | GLYMA06G38440 |
| *Gm.NAC084* | Glyma.19G108800 | | GLYMA19G28476 |
| *Gm.NAC085* | Glyma.16G043200 | | GLYMA16G04740 |
| *Gm.NAC086* | Glyma.16G151500 | | GLYMA16G26740 |
| *Gm.NAC087* | Glyma.02G070000 | | GLYMA02G07700 |
| *Gm.NAC088* | Glyma.03G179600 | | GLYMA03G33690 |
| *Gm.NAC089* | Glyma.19G180300 | | GLYMA19G36421 |
| *Gm.NAC090* | Glyma.12G171600 | | GLYMA12G29360 |
| *Gm.NAC091* | Glyma.13G327600 | | GLYMA13G40251 |
| *Gm.NAC092* | Glyma.19G109100 | | GLYMA19G28515 |
| *Gm.NAC093* | Glyma.16G042900 | | GLYMA16G04720 |
| *Gm.NAC094* | Glyma.16G152100 | | GLYMA16G26810 |
| *Gm.NAC095* | Glyma.02G070600 | | GLYMA02G07760 |
| *Gm.NAC096* | Glyma.17G101500 | | GLYMA17G10970 |
| *Gm.NAC097* | Glyma.05G025500 | | GLYMA05G00930 |
| *Gm.NAC098* | Glyma.06G195500 | | GLYMA06G21020 |
| *Gm.NAC099* | Glyma.04G167200 | | GLYMA04G33270 |
| *Gm.NAC100* | Glyma.13G274300 | | GLYMA13G34950 |
| *Gm.NAC101* | Glyma.12G226500 | | GLYMA12G35530 |
| *Gm.NAC102* | Glyma.12G161700 | | GLYMA12G26190 |
| *Gm.NAC103* | Glyma.06G236000 | | GLYMA06G35660 |
| *Gm.NAC104* | Glyma.06G080200 | | GLYMA06G08440 |
| *Gm.NAC105* | Glyma.04G078600 | | GLYMA04G08320 |
| *Gm.NAC106* | Glyma.09G184100 | | GLYMA09G31650 |
| *Gm.NAC107* | Glyma.07G092000 | | GLYMA07G10240 |
| *Gm.NAC108* | Glyma.08G041500 | | GLYMA08G04610 |
| *Gm.NAC109* | Glyma.05G234200 | | GLYMA05G35090 |
| *Gm.NAC110* | Glyma.19G024500 | | GLYMA19G02850 |
| *Gm.NAC111* | Glyma.13G062000 | | GLYMA13G05540 |
| *Gm.NAC112* | Glyma.18G261300 | | GLYMA18G49620 |
| *Gm.NAC113* | Glyma.09G235700 | | GLYMA09G37050 |
| *Gm.NAC114* | Glyma.19G165600 | | GLYMA19G34881 |
| *Gm.NAC115* | Glyma.03G164200 | | GLYMA03G32116 |
| *Gm.NAC116* | Glyma.15G254000 | | GLYMA15G40510 |
| *Gm.NAC117* | Glyma.08G173400 | | GLYMA08G18470 |
| *Gm.NAC118* | Glyma.06G014900 | | GLYMA06G01741 |
| *Gm.NAC119* | Glyma.04G014900 | | GLYMA04G01651 |
| *Gm.NAC120* | Glyma.08G161300 | | GLYMA08G17140 |
| *Gm.NAC121* | Glyma.15G266500 | | GLYMA15G42050 |
| *Gm.NAC122* | Glyma.19G259500 | | GLYMA19G44890 |
| *Gm.NAC123* | Glyma.19G259700 | | GLYMA19G44910 |
| *Gm.NAC124* | Glyma.16G019400 | | GLYMA16G02200 |
| *Gm.NAC125* | Glyma.07G050600 | | GLYMA07G05660 |
| *Gm.NAC126* | Glyma.12G003200 | | GLYMA12G00540 |
| *Gm.NAC127* | Glyma.09G233600 | | GLYMA09G36820 |
| *Gm.NAC128* | Glyma.20G175500 | | GLYMA20G31551 |
| *Gm.NAC129* | Glyma.10G216400 | | GLYMA10G36050 |
| *Gm.NAC130* | Glyma.17G138100 | | GLYMA17G14700 |
| *Gm.NAC131* | Glyma.05G055900 | | GLYMA05G04250 |
| *Gm.NAC132* | Glyma.12G004900 | | GLYMA12G00760 |
| *Gm.NAC133* | Glyma.09G231700 | | GLYMA09G36600 |
| *Gm.NAC134* | Glyma.06G318900 | | GLYMA06G47680 |
| *Gm.NAC135* | Glyma.04G119500 | | GLYMA04G13660 |
| *Gm.NAC136* | Glyma.16G130200 | | GLYMA16G24200 |
| *Gm.NAC137* | Glyma.02G050100 | | GLYMA02G05620 |
| *Gm.NAC138* | Glyma.11G075400 | | GLYMA11G07990 |
| *Gm.NAC139* | Glyma.01G167900 | | GLYMA01G37310 |

|  |
| --- |
