# Supplementary material for: The essence of NAC gene family to the cultivation of drought-resistant soybean (Glycine max L. Merr.) cultivars
Source: BMC Plant Biol. 2017 Feb 28;17:55. doi: 10.1186/s12870-017-1001-y (PMC5330122; doi:10.1186/s12870-017-1001-y)
Supplement: Additional file 2: — Table of gene’s names and gene bank accession number of Arabidopsis and Rice. (DOCX 87 kb) [file 12870_2017_1001_MOESM2_ESM.docx]

| ACCE ACCESSION | DEFINITION | Also known as | Related articles |
| --- | --- | --- | --- |
| NP_192064 | NAC transcription factor [Arabidopsis thaliana]  salt resistance | anac069; F11O4.5; F11O4_5; NAC domain containing protein 69; NAC069 | [Integration of auxin and salt signals by the NAC transcription factor NTM2 during seed germination in Arabidopsis.](http://www.ncbi.nlm.nih.gov/pubmed/21450938/) Park J, et al. Plant Physiol, 2011 Jun. PMID 21450938 |
| NP_191750 | NAC transcription factor NST2 [Arabidopsis thaliana]  (NAC domain protein 66 (NAC066);  FUNCTIONS IN: transcription  activator activity,  sequence-specific DNA binding  transcription factor activity;  INVOLVED IN:  multicellular organismal  development,  anther dehiscence, secondary  cell wall biogenesis | ANAC066; NAC domain protein 66;  NAC SECONDARY WALL THICKENING  PROMOTING FACTOR2; NAC066; NST2 | The NAC transcription factors NST1 and NST2 of Arabidopsis regulate secondary wall thickenings and are required for anther dehiscence.  *Mitsuda N, Seki M, Shinozaki K, Ohme-Takagi M. Plant Cell. 2005 Nov; 17(11):2993-3006. Epub 2005 Oct 7.* |
| NP_565404 | NAC transcription factor family protein NAC036 [Arabidopsis  thaliana]  ANAC036 gene indicated that  this gene was strongly  expressed in leaves  ANAC036 and its orthologues  are involved in the growth  of leaf cells. | anac036; F6P23.7; F6P23_7;  NAC domain containing protein 36; NAC036 | Overexpression of the NAC transcription factor family gene ANAC036 results in a dwarf phenotype in Arabidopsis thaliana.  *Kato H, Motomura T, Komeda Y, Saito T, Kato A. J Plant Physiol. 2010 May 1; 167(7):571-7. Epub 2009 Dec 4.* |
| NP_182200 | NAC transcription factor NST1  [Arabidopsis thaliana] | ANAC043;  Arabidopsis NAC domain containing protein 43;  EMB2301; EMBRYO DEFECTIVE 2301;  F19D11.5; NAC SECONDARY WALL  THICKENING  PROMOTING FACTOR1; NST1 | NAC transcription factors NST1 and NST3 regulate pod shattering in a partially redundant manner by promoting secondary wall formation after the establishment of tissue identity.  *Mitsuda N, Ohme-Takagi M. Plant J. 2008 Dec; 56(5):768-78. Epub 2008 Jul 23*  Two NAC domain transcription factors, SND1 and NST1, function redundantly in regulation of secondary wall synthesis in fibers of Arabidopsis.  *Zhong R, Richardson EA, Ye ZH. Planta. 2007 May; 225(6):1603-11. Epub 2007 Mar 1*  NAC transcription factors, NST1 and NST3, are key regulators of the formation of secondary walls in woody tissues of Arabidopsis.  *Mitsuda N, Iwase A, Yamamoto H, Yoshida M, Seki M, Shinozaki K, Ohme-Takagi M. Plant Cell. 2007 Jan; 19(1):270-80. Epub 2007 Jan 19* |
| NP_564966 | NAC transcription  factor protein family  [Arabidopsis thaliana] | activated by AP3/PI;  ACTIVATED BY AP3/PI;  ANAC029;  Arabidopsis NAC domain containing protein 29;  ATNAP; F10D13.14;  F10D13_14; NAC-like; NAC-LIKE; NAP | AtNAP, a NAC family transcription factor, has an important role in leaf senescence.  *Guo Y, Gan S. Plant J. 2006 May; 46(4):601-12.* |
| NP_174554 | NAC Domain transcription  factor  SND1 [Arabidopsis thaliana] | ANAC012; F6N18.15; F6N18_15;  NAC domain containing protein 12;  NAC SECONDARY WALL  THICKENING PROMOTING 3;  NAC012; NST3;  SECONDARY WALL-ASSOCIATED  NAC DOMAIN 1; SND1 | [ANAC012, a member of the plant-specific NAC transcription factor family, negatively regulates xylary fiber development in Arabidopsis thaliana.](http://www.ncbi.nlm.nih.gov/pubmed/17565617/) Ko JH, et al. Plant J, 2007 Jun. PMID 17565617  [The MYB46 transcription factor is a direct target of SND1 and regulates secondary wall biosynthesis in Arabidopsis.](http://www.ncbi.nlm.nih.gov/pubmed/17890373/) Zhong R, et al. Plant Cell, 2007 Sep. PMID 17890373, |
| AEE85335 | NAC transcription factor  RD26 [Arabidopsis thaliana] | ANAC072;  Arabidopsis NAC domain containing protein 72;  F27G19.10; F27G19_10; RD26;  RESPONSIVE TO DESICCATION 26 | [Co-expression of the stress-inducible zinc finger homeodomain ZFHD1 and NAC transcription factors enhances expression of the ERD1 gene in Arabidopsis.](http://www.ncbi.nlm.nih.gov/pubmed/17233795/) Tran LS, et al. Plant J, 2007 Jan. PMID 17233795 |
| Q8GY42 | RecName: Full=NAC  transcription factor 25; Short=AtNAC025 | anac025; F11P17.16; F11P17_16;  NAC domain containing protein 25; NAC025 | - A cis regulatory element in the TAPNAC promoter directs tapetal gene expression.   *Alvarado VY, Tag A, Thomas TL. Plant Mol Biol. 2011 Jan; 75(1-2):129-39. Epub 2010 Nov 24* |
| NP_188169 | ATAF-like NAC-domain transcription factor [Arabidopsis thaliana] | ANAC055; ATNAC3; NAC domain containing protein 3; NAC domain containing protein 55; NAC-DOMAIN ENCODING GENE; NAC055; NAC3 | [The Arabidopsis RING finger E3 ligase RHA2a is a novel positive regulator of abscisic acid signaling during seed germination and early seedling development.](http://www.ncbi.nlm.nih.gov/pubmed/19286935/) Bu Q, et al. Plant Physiol, 2009 May. PMID 19286935,  [Role of the Arabidopsis thaliana NAC transcription factors ANAC019 and ANAC055 in regulating jasmonic acid-signaled defense responses.](http://www.ncbi.nlm.nih.gov/pubmed/18427573/) Bu Q, et al. Cell Res, 2008 Jul. PMID 18427573 |
| NP_568414 | membrane-tethered transcription factor ANAC089 [Arabidopsis thaliana] | anac089; NAC domain containing protein 89; NAC089; T6G21.9 | [The membrane-associated transcription factor NAC089 controls ER-stress-induced programmed cell death in plants.](http://www.ncbi.nlm.nih.gov/pubmed/24675811/) Yang ZT, et al. PLoS Genet, 2014 Mar. PMID 24675811  [A membrane-tethered transcription factor ANAC089 negatively regulates floral initiation in Arabidopsis thaliana.](http://www.ncbi.nlm.nih.gov/pubmed/21046321/) Li J, et al. Sci China Life Sci, 2010 Nov. PMID 21046321 |
| NP_198777 | NAC-domain transcription factor [Arabidopsis thaliana] | ANAC092; Arabidopsis NAC domain containing protein 92; ATNAC2; ATNAC6; MIJ24.11; MIJ24_11; NAC domain containing protein 2; NAC domain containing protein 6; NAC2; NAC6; ORE1; ORESARA 1 | [[Functions of ANAC092 involved in regulation of anther development in Arabidopsis thaliana].](http://www.ncbi.nlm.nih.gov/pubmed/23853363/) Li J, et al. Yi Chuan, 2013 Jul. PMID 23853363  [NAC transcription factor ORE1 and senescence-induced BIFUNCTIONAL NUCLEASE1 (BFN1) constitute a regulatory cascade in Arabidopsis.](http://www.ncbi.nlm.nih.gov/pubmed/23340744/) Matallana-Ramirez LP, et al. Mol Plant, 2013 Sep. PMID 23340744  [A gene regulatory network controlled by the NAC transcription factor ANAC092/AtNAC2/ORE1 during salt-promoted senescence.](http://www.ncbi.nlm.nih.gov/pubmed/20113437/) Balazadeh S, et al. Plant J, 2010 Apr. PMID 20113437  [The delayed leaf senescence mutants of Arabidopsis, ore1, ore3, and ore9 are tolerant to oxidative stress.](http://www.ncbi.nlm.nih.gov/pubmed/15295076/) Woo HR, et al. Plant Cell Physiol, 2004 Jul. PMID 15295076  [ORE1 balances leaf senescence against maintenance by antagonizing G2-like-mediated transcription.](http://www.ncbi.nlm.nih.gov/pubmed/23459204/) Rauf M, et al. EMBO Rep, 2013 Apr. PMID 23459204, |
| NP_201044 | NAC-domain transcription factor [Arabidopsis thaliana] | ANAC101; MMI9.6; MMI9_6; NAC-domain protein 101; NAC101; VASCULAR-RELATED NAC-DOMAIN 6; VND6 | [VASCULAR-RELATED NAC-DOMAIN6 and VASCULAR-RELATED NAC-DOMAIN7 effectively induce trans differentiation into xylem vessel elements under control of an induction system.](http://www.ncbi.nlm.nih.gov/pubmed/20488898/) Yamaguchi M, et al. Plant Physiol, 2010 Jul. PMID 20488898  [Arabidopsis VASCULAR-RELATED NAC-DOMAIN6 directly regulates the genes that govern programmed cell death and secondary wall formation during xylem differentiation.](http://www.ncbi.nlm.nih.gov/pubmed/20952636/) Ohashi-Ito K, et al. Plant Cell, 2010 Oct. PMID 20952636 |
| NP_00111912 | NAC transcription factor-like 9 [Arabidopsis thaliana] | F8D20.90; F8D20_90;  NAC transcription factor-like 9; NTL9 | [Regulation of leaf senescence by NTL9-mediated osmotic stress signaling in Arabidopsis.](http://www.ncbi.nlm.nih.gov/pubmed/18443413/) Yoon HK, et al. Mol Cells, 2008 May 31. PMID 18443413  [The Pseudomonas syringae type III effector HopD1 suppresses effector-triggered immunity, localizes to the endoplasmic reticulum, and targets the Arabidopsis transcription factor NTL9.](http://www.ncbi.nlm.nih.gov/pubmed/24329768/) Block A, et al. New Phytol, 2014 Mar. PMID 24329768 |
| NP_849817 | transcription factor NAC1 [Arabidopsis thaliana] | anac021; ANAC022; Arabidopsis NAC domain containing protein 21; Arabidopsis NAC domain containing protein 22; F14J16.32; NAC domain containing protein 1; NAC1 | [In silico analysis on structure and DNA binding mode of AtNAC1, a NAC transcription factor from Arabidopsis thaliana.](http://www.ncbi.nlm.nih.gov/pubmed/24570356/) Zhu Q, et al. J Mol Model, 2014 Mar. PMID 24570356  [Expression of NAC1 up-stream regulatory region and its relationship to the lateral root initiation induced by gibberellins and auxins.](http://www.ncbi.nlm.nih.gov/pubmed/17172049/) Wang Y, et al. Sci China C Life Sci, 2006 Oct. PMID 17172049  [Arabidopsis NAC1 transduces auxin signal downstream of TIR1 to promote lateral root development.](http://www.ncbi.nlm.nih.gov/pubmed/11114891/) Xie Q, et al. Genes Dev, 2000 Dec 1. PMID 11114891 |
| Q9SK55 | RecName: Full=Transcription factor JUNGBRUNNEN 1; AltName: Full=NAC  domain-containing protein 42; Short=ANAC042. | anac042; F23E6.1; F23E6_1;  NAC domain containing protein 42; NAC042 | Identification and characterization of ANAC042, a transcription factor family gene involved in the regulation of camalexin biosynthesis in Arabidopsis.  *Saga H, Ogawa T, Kai K, Suzuki H, Ogata Y, Sakurai N, Shibata D, Ohta D. Mol Plant Microbe Interact. 2012 May; 25(5):684-96.* |
| A8MQY1 | RecName: Full=NAC domain-containing protein 68; Short=ANAC068; AltName: Full=Protein NAC WITH TRANSMEMBRANE MOTIF 1.  Encodes a membrane-bound NAC (for NAM, ATAF1/2, CUC2) transcription factor, designated NTM1 (for NAC with transmembrane motif1). NTM1 regulates cell division in Arabidopsis. | ANAC068; Arabidopsis NAC domain containing protein 68; F11O4.4; F11O4_4; NAC with transmembrane motif1; NTM1 |  |
| AEE32863 | NAC domain-containing protein 18 [Arabidopsis thaliana] | ANAC018; Arabidopsis NAC domain containing protein 18; ATNAM; F14G24.15;  F14G24_15; NAC DOMAIN PROTEIN NAM; NAC-REGULATED SEED MORPHOLOGY 2; NAM; NARS2; NO APICAL MERISTEM |  |
| NP_973954 | protein NAC 014 [Arabidopsis thaliana]  FUNCTIONS IN: sequence-specific DNA binding transcription factor activity; INVOLVED IN: multicellular organismal development, regulation of transcription; LOCATED IN: cellular_component | NTM1; NAC 014; NAC014; T9L6.13; T9L6_13 |  |
| NP_001078343 | NAC with transmembrane motif1 [Arabidopsis thaliana] | ANAC068; Arabidopsis NAC domain containing protein 68; F11O4.4; F11O4_4; NAC with transmembrane motif1; NTM1 | [Proteomic identification of differentially expressed proteins in Arabidopsis mutant ntm1-D with disturbed cell division.](http://www.ncbi.nlm.nih.gov/pubmed/18319616/) Lee KH, et al. Mol Cells, 2008 Feb 29. PMID 18319616 |
| NP_001154602 | NAC domain containing protein 50 [Arabidopsis thaliana] | ANAC050; NAC domain containing protein 50; NAC050 |  |
| NP_974800 | NAC domain containing protein 87 [Arabidopsis thaliana] | Arabidopsis NAC domain containing protein 87; MRG7.23; MRG7_23 | [An NAC transcription factor controls ethylene-regulated cell expansion in flower petals.](http://www.ncbi.nlm.nih.gov/pubmed/23933991/) Pei H, et al. Plant Physiol, 2013 Oct. PMID 23933991 |
| NP_568182 | NAC domain containing protein 80 [Arabidopsis thaliana] | ANAC079; ANAC080; Arabidopsis NAC domain containing protein 79; ATNAC4; MBK20.13; MBK20_13; NAC domain containing protein 80; NAC080 |  |
| NP_196822 | NAC domain containing protein 83 [Arabidopsis thaliana] | ANAC083; NAC domain containing protein 83; NAC083; T19L5.140; T19L5_140; VND-interacting 2; VNI2 | [The Arabidopsis NAC transcription factor VNI2 integrates abscisic acid signals into leaf senescence via the COR/RD genes.](http://www.ncbi.nlm.nih.gov/pubmed/21673078/) Yang SD, et al. Plant Cell, 2011 Jun. PMID 21673078.  [VND-INTERACTING2, a NAC domain transcription factor, negatively regulates xylem vessel formation in Arabidopsis.](http://www.ncbi.nlm.nih.gov/pubmed/20388856/) Yamaguchi M, et al. Plant Cell, 2010 Apr. PMID 20388856 |
| NP_197331 | NAC domain containing protein 88 [Arabidopsis thaliana] | anac088; F20L16.20; NAC domain containing protein 88; NAC088 |  |
| NP_195339 | NAC domain containing protein 76 [Arabidopsis thaliana]  Encodes a NAC-domain transcription factor. Expressed in the vascular  tissue | ANAC076; F23E13.50; F23E13_50; NAC domain containing protein 76; NAC076; VASCULAR-RELATED NAC-DOMAIN 2; VND2 | [VND-INTERACTING2, a NAC domain transcription factor, negatively regulates xylem vessel formation in Arabidopsis.](http://www.ncbi.nlm.nih.gov/pubmed/20388856/) Yamaguchi M, et al. Plant Cell, 2010 Apr. PMID 20388856.  [SND1, a NAC domain transcription factor, is a key regulator of secondary wall synthesis in fibers of Arabidopsis.](http://www.ncbi.nlm.nih.gov/pubmed/17114348/) Zhong R, et al. Plant Cell, 2006 Nov. PMID 17114348. |
| NP_188469 | NAC domain containing protein 58 [Arabidopsis thaliana] | anac058; NAC domain containing protein 58; NAC058 | modulators of ABA-mediated germination potential  The TRANSPLANTA collection of Arabidopsis lines: a resource for functional analysis of transcription factors based on their conditional overexpression.  [Coego A](http://www.ncbi.nlm.nih.gov/pubmed/?term=Coego%20A%5BAuthor%5D&cauthor=true&cauthor_uid=24456507)^1^, [Brizuela E](http://www.ncbi.nlm.nih.gov/pubmed/?term=Brizuela%20E%5BAuthor%5D&cauthor=true&cauthor_uid=24456507), [Castillejo P](http://www.ncbi.nlm.nih.gov/pubmed/?term=Castillejo%20P%5BAuthor%5D&cauthor=true&cauthor_uid=24456507), [Ruíz S](http://www.ncbi.nlm.nih.gov/pubmed/?term=Ru%C3%ADz%20S%5BAuthor%5D&cauthor=true&cauthor_uid=24456507), [Koncz C](http://www.ncbi.nlm.nih.gov/pubmed/?term=Koncz%20C%5BAuthor%5D&cauthor=true&cauthor_uid=24456507), [del Pozo JC](http://www.ncbi.nlm.nih.gov/pubmed/?term=del%20Pozo%20JC%5BAuthor%5D&cauthor=true&cauthor_uid=24456507), [Piñeiro M](http://www.ncbi.nlm.nih.gov/pubmed/?term=Pi%C3%B1eiro%20M%5BAuthor%5D&cauthor=true&cauthor_uid=24456507), [Jarillo JA](http://www.ncbi.nlm.nih.gov/pubmed/?term=Jarillo%20JA%5BAuthor%5D&cauthor=true&cauthor_uid=24456507), [Paz-Ares J](http://www.ncbi.nlm.nih.gov/pubmed/?term=Paz-Ares%20J%5BAuthor%5D&cauthor=true&cauthor_uid=24456507), [León J](http://www.ncbi.nlm.nih.gov/pubmed/?term=Le%C3%B3n%20J%5BAuthor%5D&cauthor=true&cauthor_uid=24456507); [TRANSPLANTA Consortium](http://www.ncbi.nlm.nih.gov/pubmed/?term=TRANSPLANTA%20Consortium%5BCorporate%20Author%5D).  [Plant J.](http://www.ncbi.nlm.nih.gov/pubmed/?term=ANAC058) 2014 Mar;77(6):944-53. doi: 10.1111/tpj.12443. Epub 2014 Feb 27 |
| NP_191215 | NAC domain containing protein 65 [Arabidopsis thaliana] | anac065; NAC domain containing protein 65; NAC065 |  |
| NP_198798 | NAC domain containing protein 94 [Arabidopsis thaliana] | anac094; MKM21.110; MKM21_110; NAC domain containing protein 94; NAC094 |  |
| NP_171727 | NAC domain-containing protein 5 [Arabidopsis thaliana] | anac005; NAC domain containing protein 5; NAC005; T6A9.20 |  |
| NP_001119074 | NAC domain containing protein 74 [Arabidopsis thaliana] | anac074; F20O9.220; F20O9_220; NAC domain containing protein 74; NAC074 |  |
| NP_001118568 | NAC domain containing protein 47 [Arabidopsis thaliana] | anac047; NAC domain containing protein 47; NAC047; T11I18.18; T11I18_18 | [NAC family proteins NARS1/NAC2 and NARS2/NAM in the outer integument regulate embryogenesis in Arabidopsis.](http://www.ncbi.nlm.nih.gov/pubmed/18849494/) Kunieda T, et al. Plant Cell, 2008 Oct. PMID 18849494, |
| NP_186970 | NAC domain containing protein 45 [Arabidopsis thaliana] | anac045; NAC domain containing protein 45; NAC045; T17B22.11; T17B22_11 | [Plant development. Arabidopsis NAC45/86 direct sieve element morphogenesis culminating in enucleation.](http://www.ncbi.nlm.nih.gov/pubmed/25081480/) Furuta KM, et al. Science, 2014 Aug 22. PMID 25081480 |
| NP_001118435 | NAC domain containing protein 41 [Arabidopsis thaliana] | ANAC041; F4P9.25; F4P9_25; NAC domain containing protein 41; NAC041 | [Transcription factors that directly regulate the expression of CSLA9 encoding mannan synthase in Arabidopsis thaliana.](http://www.ncbi.nlm.nih.gov/pubmed/24243147/) Kim WC, et al. Plant Mol Biol, 2014 Mar. PMID 24243147 |
| NP_199895 | NAC domain containing protein 97 [Arabidopsis thaliana] | anac097; K7B16.4; K7B16_4; NAC domain containing protein 97; NAC097 |  |
| NP_171726 | NAC domain-containing protein 4 [Arabidopsis thaliana] | ANAC004; NAC domain containing protein 4; NAC004; T6A9.19 |  |
| NP_001078551 | NAC domain containing protein 82 [Arabidopsis thaliana] | anac082; NAC domain containing protein 82; NAC082; T5E8.130; T5E8_130; VND-interacting 1; VNI1 |  |
| NP_001078103 | NAC domain containing protein 48 [Arabidopsis thaliana] | anac048; NAC domain containing protein 48; NAC048; T27C4.6; T27C4_6 |  |
| NP_175835 | NAC domain containing protein 20 [Arabidopsis thaliana] | ANAC020; F20D21.15; F20D21_15; NAC domain containing protein 20; NAC020 |  |
| NP_974272 | NAC domain containing protein 50 [Arabidopsis thaliana] | ANAC050; NAC domain containing protein 50; NAC050 |  |
| NP_850054 | NAC domain containing protein 38 [Arabidopsis thaliana] | ANAC038; ANAC039; Arabidopsis NAC domain containing protein 39; NAC domain containing protein 38; NAC038; T28I24.16; T28I24_16 |  |
| NP_187057 | NAC domain containing protein 47 [Arabidopsis thaliana] | anac047; NAC domain containing protein 47; NAC047; T11I18.18; T11I18_18 |  |
| NP_568292 | NAC domain containing protein 84 [Arabidopsis thaliana] | anac084; MAC12.3; MAC12_3; NAC domain containing protein 84; NAC084 |  |
| NP_564771 | NAC domain containing protein 25 [Arabidopsis thaliana] | anac025; F11P17.16; F11P17_16; NAC domain containing protein 25; NAC025 |  |
| NP_564440 | NAC domain containing protein 17 [Arabidopsis thaliana] | anac017; F12G12.30; NAC domain containing protein 17; NAC017 |  |
| NP_566376 | NAC domain containing protein 53 [Arabidopsis thaliana] | anac053; NAC domain containing protein 53; NAC053 | [The NAC-like gene ANTHER INDEHISCENCE FACTOR acts as a repressor that controls anther dehiscence by regulating genes in the jasmonate biosynthesis pathway in Arabidopsis.](http://www.ncbi.nlm.nih.gov/pubmed/24323506/) Shih CF, et al. J Exp Bot, 2014 Feb. PMID 24323506 |
| NP_566374 | NAC domain containing protein 50 [Arabidopsis thaliana] | ANAC050; NAC domain containing protein 50; NAC050 |  |
| NP_197630 | NAC domain-containing protein [Arabidopsis thaliana] | anac090; MWD9.18; MWD9_18; NAC domain containing protein 90; NAC090 |  |
| NP_200473 | NAC domain containing protein 99 [Arabidopsis thaliana] | anac099; MIK19.7; MIK19_7; NAC domain containing protein 99; NAC099 |  |
| NP_200951 | NAC domain containing protein 100 [Arabidopsis thaliana] | ANAC100; ATNAC5; MFB13.6; MFB13_6; NAC domain containing protein 100; NAC100 | [An NAC transcription factor controls ethylene-regulated cell expansion in flower petals.](http://www.ncbi.nlm.nih.gov/pubmed/23933991/) Pei H, et al. Plant Physiol, 2013 Oct. PMID 23933991 |
| NP_201431 | NAC domain containing protein [Arabidopsis thaliana] | ANAC105; Arabidopsis NAC domain containing protein 105; K1L20.8; K1L20_8; NAC domain containing protein 105; NAC105; VASCULAR-RELATED NAC-DOMAIN 3; VND3 | [VND-INTERACTING2, a NAC domain transcription factor, negatively regulates xylem vessel formation in Arabidopsis.](http://www.ncbi.nlm.nih.gov/pubmed/20388856/) Yamaguchi M, et al. Plant Cell, 2010 Apr. PMID 20388856, |
| NP_197228 | NAC domain containing protein 86 [Arabidopsis thaliana] | anac086; MKP11.11; MKP11_11; NAC domain containing protein 86; NAC086 | [Plant development. Arabidopsis NAC45/86 direct sieve element morphogenesis culminating in enucleation.](http://www.ncbi.nlm.nih.gov/pubmed/25081480/) Furuta KM, et al. Science, 2014 Aug 22. PMID 25081480 |
| NP_201211 | NAC domain containing protein 103 [Arabidopsis thaliana] | anac103; MHJ24.4; MHJ24_4; NAC domain containing protein 103; NAC103 | [The plant-specific transcription factor gene NAC103 is induced by bZIP60 through a new cis-regulatory element to modulate the unfolded protein response in Arabidopsis.](http://www.ncbi.nlm.nih.gov/pubmed/23869562/) Sun L, et al. Plant J, 2013 Oct. PMID 23869562 |
| NP_198925 | NAC domain containing protein 95 [Arabidopsis thaliana] | anac095; MEE6.16; MEE6_16; NAC domain containing protein 95; NAC095 | [Wide-scale screening of T-DNA lines for transcription factor genes affecting male gametophyte development in Arabidopsis.](http://www.ncbi.nlm.nih.gov/pubmed/22101548/) Reňák D, et al. Sex Plant Reprod, 2012 Mar. PMID 22101548 |
| NP_199471 | NAC domain containing protein 96 [Arabidopsis thaliana] | anac096; F10E10.6; F10E10_6; NAC domain containing protein 96; NAC096 | [The Arabidopsis NAC transcription factor ANAC096 cooperates with bZIP-type transcription factors in dehydration and osmotic stress responses.](http://www.ncbi.nlm.nih.gov/pubmed/24285786/) Xu ZY, et al. Plant Cell, 2013 Nov. PMID 24285786 |
| NP_193532 | NAC domain containing protein 71 [Arabidopsis thaliana] | anac071; NAC domain containing protein 71; NAC071; T6K21.160; T6K21_160 | [Spatially selective hormonal control of RAP2.6L and ANAC071 transcription factors involved in tissue reunion in Arabidopsis.](http://www.ncbi.nlm.nih.gov/pubmed/21911380/) Asahina M, et al. Proc Natl Acad Sci U S A, 2011 Sep 20. PMID 21911380 |
| NP_188170 | NAC domain containing protein 2 [Arabidopsis thaliana] | ANAC056; Arabidopsis NAC domain containing protein 56; ATNAC2; NAC domain containing protein 2; NAC-REGULATED SEED MORPHOLOGY 1; NAC2; NARS1 | [AtNAC2, a transcription factor downstream of ethylene and auxin signaling pathways, is involved in salt stress response and lateral root development.](http://www.ncbi.nlm.nih.gov/pubmed/16359384/) He XJ, et al. Plant J, 2005 Dec. PMID 16359384  [NAC family proteins NARS1/NAC2 and NARS2/NAM in the outer integument regulate embryogenesis in Arabidopsis.](http://www.ncbi.nlm.nih.gov/pubmed/18849494/) Kunieda T, et al. Plant Cell, 2008 Oct. PMID 18849494 |
| NP_186809 | NAC domain containing protein 44 [Arabidopsis thaliana] | anac044; F4P13.14; F4P13_14; NAC domain containing protein 44; NAC044 |  |
| NP_190015 | NAC domain containing protein 60 [Arabidopsis thaliana] | anac060; NAC domain containing protein 60; NAC060 | [The ABI4-induced Arabidopsis ANAC060 transcription factor attenuates ABA signaling and renders seedlings sugar insensitive when present in the nucleus.](http://www.ncbi.nlm.nih.gov/pubmed/24625790/) Li P, et al. PLoS Genet, 2014 Mar. PMID 24625790 |
| NP_188400 | NAC domain containing protein 57 [Arabidopsis thaliana] | anac057; NAC domain containing protein 57; NAC057 |  |
| NP_187093 | NAC domain containing protein 49 [Arabidopsis thaliana] | anac049; NAC domain containing protein 49; NAC049; T27C4.7; T27C4_7 |  |
| NP_187056 | NAC domain containing protein 46 [Arabidopsis thaliana] | anac046; NAC domain containing protein 46; NAC046; T11I18.17; T11I18_17 |  |
| NP_180906 | NAC domain containing protein 41 [Arabidopsis thaliana] | ANAC041; F4P9.25; F4P9_25; NAC domain containing protein 41; NAC041 | [Transcription factors that directly regulate the expression of CSLA9 encoding mannan synthase in Arabidopsis thaliana.](http://www.ncbi.nlm.nih.gov/pubmed/24243147/) Kim WC, et al. Plant Mol Biol, 2014 Mar. PMID 24243147 |
| NP_180019 | NAC domain containing protein 38 [Arabidopsis thaliana] | ANAC038; ANAC039; Arabidopsis NAC domain containing protein 39; NAC domain containing protein 38; NAC038; T28I24.16; T28I24_16 |  |
| NP_177869 | NAC domain containing protein 32 [Arabidopsis thaliana] | anac032; NAC domain containing protein 32; NAC032; T5M16.4; T5M16_4 | [NAC family proteins NARS1/NAC2 and NARS2/NAM in the outer integument regulate embryogenesis in Arabidopsis.](http://www.ncbi.nlm.nih.gov/pubmed/18849494/) Kunieda T, et al. Plant Cell, 2008 Oct. PMID 18849494 |
| NP_176457 | NAC domain containing protein 26 [Arabidopsis thaliana] | Arabidopsis NAC domain containing protein 26; F23N19.6; F23N19_6; VASCULAR RELATED NAC-DOMAIN PROTEIN 5; VND5 |  |
| NP_172690 | NAC domain-containing protein 7 [Arabidopsis thaliana] | ANAC007; EMB2749; EMBRYO DEFECTIVE 2749; NAC 007; NAC007; T28K15.1; T28K15_1; VASCULAR RELATED NAC-DOMAIN PROTEIN 4; VND4 |  |
| NP_175697 | NAC domain-containing protein 19 [Arabidopsis thaliana] | ANAC019; F14G24.16; F14G24_16; NAC domain containing protein 19; NAC019 | [DNA binding by the plant-specific NAC transcription factors in crystal and solution: a firm link to WRKY and GCM transcription factors.](http://www.ncbi.nlm.nih.gov/pubmed/22455904/) Welner DH, et al. Biochem J, 2012 Jun 15. PMID 22455904  [The Arabidopsis thaliana NAC transcription factor family: structure-function relationships and determinants of ANAC019 stress signalling.](http://www.ncbi.nlm.nih.gov/pubmed/19995345/) Jensen MK, et al. Biochem J, 2010 Feb 9. PMID 19995345  [Role of the Arabidopsis thaliana NAC transcription factors ANAC019 and ANAC055 in regulating jasmonic acid-signaled defense responses.](http://www.ncbi.nlm.nih.gov/pubmed/18427573/) Bu Q, et al. Cell Res, 2008 Jul. PMID 18427573 |
| NP_176766 | domain containing protein 28 [Arabidopsis thaliana] | anac028; F12P19.8; F12P19_8; NAC domain containing protein 28; NAC028 |  |
| AEE86626 | NAC domain containing protein 76 [Arabidopsis thaliana] | ANAC076; F23E13.50; F23E13_50; NAC domain containing protein 76; NAC076; VASCULAR-RELATED NAC-DOMAIN 2; VND2 | [VND-INTERACTING2, a NAC domain transcription factor, negatively regulates xylem vessel formation in Arabidopsis.](http://www.ncbi.nlm.nih.gov/pubmed/20388856/) Yamaguchi M, et al. Plant Cell, 2010 Apr. PMID 20388856  [SND1, a NAC domain transcription factor, is a key regulator of secondary wall synthesis in fibers of Arabidopsis.](http://www.ncbi.nlm.nih.gov/pubmed/17114348/) Zhong R, et al. Plant Cell, 2006 Nov. PMID 17114348 |
| AEE85605 | NAC domain containing protein 75 [Arabidopsis thaliana] | anac075; F17A13.50; F17A13_50; NAC domain containing protein 75; NAC075 |  |
| AEE82036 | NAC domain containing protein 67 [Arabidopsis thaliana] | anac067; F11O4.3; F11O4_3; NAC domain containing protein 67; NAC067 |  |
| AEE76092 | NAC domain containing protein 58 [Arabidopsis thaliana] | anac058; NAC domain containing protein 58; NAC058 |  |
| AEE76000 | NAC domain containing protein 57 [Arabidopsis thaliana] | anac057; NAC domain containing protein 57; NAC057 |  |
| AEE74917 | NAC domain containing protein 53 [Arabidopsis thaliana] | anac053; NAC domain containing protein 53; NAC053 | [The NAC-like gene ANTHER INDEHISCENCE FACTOR acts as a repressor that controls anther dehiscence by regulating genes in the jasmonate biosynthesis pathway in Arabidopsis.](http://www.ncbi.nlm.nih.gov/pubmed/24323506/) Shih CF, et al. J Exp Bot, 2014 Feb. PMID 24323506  [A NAC transcription factor NTL4 promotes reactive oxygen species production during drought-induced leaf senescence in Arabidopsis.](http://www.ncbi.nlm.nih.gov/pubmed/22313226/) Lee S, et al. Plant J, 2012 Jun. PMID 22313226 |
| AEE74914 | NAC domain containing protein 50 [Arabidopsis thaliana] | ANAC050; NAC domain containing protein 50; NAC050 |  |
| AEE74033 | NAC domain containing protein 47 [Arabidopsis thaliana] | anac047; NAC domain containing protein 47; NAC047; T11I18.18; T11I18_18 | [NAC family proteins NARS1/NAC2 and NARS2/NAM in the outer integument regulate embryogenesis in Arabidopsis.](http://www.ncbi.nlm.nih.gov/pubmed/18849494/) Kunieda T, et al. Plant Cell, 2008 Oct. PMID 18849494 |
| AEE73912 | NAC domain containing protein 45 [Arabidopsis thaliana] | anac045; NAC domain containing protein 45; NAC045; T17B22.11; T17B22_11 | [Plant development. Arabidopsis NAC45/86 direct sieve element morphogenesis culminating in enucleation.](http://www.ncbi.nlm.nih.gov/pubmed/25081480/) Furuta KM, et al. Science, 2014 Aug 22. PMID 25081480 |
| AEE34440 | NAC domain containing protein 28 [Arabidopsis thaliana] | anac028; F12P19.8; F12P19_8; NAC domain containing protein 28; NAC028 |  |
| AEE33783 | NAC domain containing protein 25 [Arabidopsis thaliana] | anac025; F11P17.16; F11P17_16; NAC domain containing protein 25; NAC025 | [A cis regulatory element in the TAPNAC promoter directs tapetal gene expression.](http://www.ncbi.nlm.nih.gov/pubmed/21107887/) Alvarado VY, et al. Plant Mol Biol, 2011 Jan. PMID 21107887 |
| AEE33081 | NAC domain containing protein 20 [Arabidopsis thaliana] | ANAC020; F20D21.15; F20D21_15; NAC domain containing protein 20; NAC020 |  |
| AEE31684 | NAC domain containing protein 17 [Arabidopsis thaliana] | anac017; F12G12.30; NAC domain containing protein 17; NAC017 |  |
| AEE27406 | NAC domain-containing protein 5 [Arabidopsis thaliana] | anac005; NAC domain containing protein 5; NAC005; T6A9.20 |  |
| AEE27405 | NAC domain-containing protein 4 [Arabidopsis thaliana] | ANAC004; NAC domain containing protein 4; NAC004; T6A9.19 |  |
| AEE27404 | NAC domain-containing protein [Arabidopsis thaliana] | ANAC003; NAC domain containing protein 3; NAC003; T6A9.17 |  |
| AEE27216 | NAC domain-containing protein 1 [Arabidopsis thaliana | ANAC001; NAC domain containing protein 1; NAC001; T25K16.1; T25K16_1 |  |
| AED98196 | NAC domain containing protein [Arabidopsis thaliana] | ANAC105; Arabidopsis NAC domain containing protein 105; K1L20.8; K1L20_8; NAC domain containing protein 105; NAC105; VASCULAR-RELATED NAC-DOMAIN 3; VND3 |  |
| AED97835 | NAC domain containing protein 103 [Arabidopsis thaliana] | anac103; MHJ24.4; MHJ24_4; NAC domain containing protein 103; NAC103 | [The plant-specific transcription factor gene NAC103 is induced by bZIP60 through a new cis-regulatory element to modulate the unfolded protein response in Arabidopsis.](http://www.ncbi.nlm.nih.gov/pubmed/23869562/) Sun L, et al. Plant J, 2013 Oct. PMID 23869562 |
| AED97468 | NAC domain containing protein 100 [Arabidopsis thaliana] | ANAC100; ATNAC5; MFB13.6; MFB13_6; NAC domain containing protein 100; NAC100 |  |
| AED96789 | NAC domain containing protein 99 [Arabidopsis thaliana] | anac099; MIK19.7; MIK19_7; NAC domain containing protein 99; NAC099 |  |
| AED95996 | NAC domain containing protein 97 [Arabidopsis thaliana] | anac097; K7B16.4; K7B16_4; NAC domain containing protein 97; NAC097 |  |
| AED92536 | NAC domain containing protein 88 [Arabidopsis thaliana] | anac088; F20L16.20; NAC domain containing protein 88; NAC088 |  |
| AED92530 | NAC domain containing protein 87 [Arabidopsis thaliana] | ANAC087 NAC domain containing protein 87 [ **Arabidopsis thaliana** (thale cress) ] | [An NAC transcription factor controls ethylene-regulated cell expansion in flower petals.](http://www.ncbi.nlm.nih.gov/pubmed/23933991/) Pei H, et al. Plant Physiol, 2013 Oct. PMID 23933991 |
| AED92040 | NAC domain containing protein 85 [Arabidopsis thaliana] | anac085; NAC domain containing protein 85; NAC085; T15N1.4 |  |
| AED91972 | NAC domain containing protein 84 [Arabidopsis thaliana] | anac084; MAC12.3; MAC12_3; NAC domain containing protein 84; NAC084 |  |
| AED91860 | NAC domain containing protein 83 [Arabidopsis thaliana] | ANAC083; NAC domain containing protein 83; NAC083; T19L5.140; T19L5_140; VND-interacting 2; VNI2 | [The Arabidopsis NAC transcription factor VNI2 integrates abscisic acid signals into leaf senescence via the COR/RD genes.](http://www.ncbi.nlm.nih.gov/pubmed/21673078/) Yang SD, et al. Plant Cell, 2011 Jun. PMID 21673078  [VND-INTERACTING2, a NAC domain transcription factor, negatively regulates xylem vessel formation in Arabidopsis.](http://www.ncbi.nlm.nih.gov/pubmed/20388856/) Yamaguchi M, et al. Plant Cell, 2010 Apr. PMID 20388856 |
| AEC08840 | NAC domain containing protein 41 [Arabidopsis thaliana] | ANAC041; F4P9.25; F4P9_25; NAC domain containing protein 41; NAC041 | [Transcription factors that directly regulate the expression of CSLA9 encoding mannan synthase in Arabidopsis thaliana.](http://www.ncbi.nlm.nih.gov/pubmed/24243147/) Kim WC, et al. Plant Mol Biol, 2014 Mar. PMID 24243147 |
| AEC07576 | domain containing protein 38 [Arabidopsis thaliana] | ANAC038; ANAC039; Arabidopsis NAC domain containing protein 39; NAC domain containing protein 38; NAC038; T28I24.16; T28I24_16 |  |
| AEC05582 | NAC domain containing protein 35 [Arabidopsis thaliana] | anac034; ANAC035; Arabidopsis NAC domain containing protein 34; LONG VEGETATIVE PHASE 1; LOV1; NAC domain containing protein 35; NAC035 |  |
| NP_850789 | NAC domain containing protein 80 [Arabidopsis thaliana] | ANAC079; ANAC080; Arabidopsis NAC domain containing protein 79; ATNAC4; MBK20.13; MBK20_13; NAC domain containing protein 80; NAC080 |  |
| AAM47975 | putative alpha NAC [Arabidopsis thaliana]  AT4G10480 Nascent polypeptide-associated complex subunit alpha-like protein 4 [ **Arabidopsis thaliana** (thale cress) ] | F7L13.60; F7L13_60 |  |
| NP_001154700 | NAC domain containing protein 82 [Arabidopsis thaliana] | anac082; NAC domain containing protein 82; NAC082; T5E8.130; T5E8_130; VND-interacting 1; VNI1 |  |
| NP_850554 | NAC domain containing protein 52 [Arabidopsis thaliana] | ANAC051; ANAC052; Arabidopsis NAC domain containing protein 51; NAC domain containing protein 52; NAC052 |  |
| NP_564823 | NAC domain containing protein 27 [Arabidopsis thaliana] | ANAC027; NAC domain containing protein 27; NAC027 |  |
| NP_566375 | NAC domain containing protein 52 [Arabidopsis thaliana] | ANAC051; ANAC052; Arabidopsis NAC domain containing protein 51; NAC domain containing protein 52; NAC052 |  |
| AEE77527 | NAC domain-containing protein 3 [Arabidopsis thaliana] | ANAC059; Arabidopsis NAC domain containing protein 59; ATNAC3; NAC domain containing protein 3; NAC3 | [ORS1, an H₂O₂-responsive NAC transcription factor, controls senescence in Arabidopsis thaliana.](http://www.ncbi.nlm.nih.gov/pubmed/21303842/) Balazadeh S, et al. Mol Plant, 2011 Mar. PMID 21303842,  [AtNAC2, a transcription factor downstream of ethylene and auxin signaling pathways, is involved in salt stress response and lateral root development.](http://www.ncbi.nlm.nih.gov/pubmed/16359384/) He XJ, et al. Plant J, 2005 Dec. PMID 16359384 |
| AEE74915 | NAC domain containing protein 52 [Arabidopsis thaliana] | ANAC051; ANAC052; Arabidopsis NAC domain containing protein 51; NAC domain containing protein 52; NAC052 | [A collection of Ds insertional mutants associated with defects in male gametophyte development and function in Arabidopsis thaliana.](http://www.ncbi.nlm.nih.gov/pubmed/19237690/) Boavida LC, et al. Genetics, 2009 Apr. PMID 19237690. |
| AEE33331 | NAC domain-containing protein 21/22 [Arabidopsis thaliana] | anac021; ANAC022; Arabidopsis NAC domain containing protein 21; Arabidopsis NAC domain containing protein 22; F14J16.32; NAC domain containing protein 1; NAC1 |  |
| AEE33330 | NAC domain-containing protein 21/22 [Arabidopsis thaliana] | anac021; ANAC022; Arabidopsis NAC domain containing protein 21; Arabidopsis NAC domain containing protein 22; F14J16.32; NAC domain containing protein 1; NAC1 | [In silico analysis on structure and DNA binding mode of AtNAC1, a NAC transcription factor from Arabidopsis thaliana.](http://www.ncbi.nlm.nih.gov/pubmed/24570356/) Zhu Q, et al. J Mol Model, 2014 Mar. PMID 24570356  [Expression of NAC1 up-stream regulatory region and its relationship to the lateral root initiation induced by gibberellins and auxins.](http://www.ncbi.nlm.nih.gov/pubmed/17172049/) Wang Y, et al. Sci China C Life Sci, 2006 Oct. PMID 17172049  [Arabidopsis NAC1 transduces auxin signal downstream of TIR1 to promote lateral root development.](http://www.ncbi.nlm.nih.gov/pubmed/11114891/) Xie Q, et al. Genes Dev, 2000 Dec 1. PMID 11114891  [SINAT5 promotes ubiquitin-related degradation of NAC1 to attenuate auxin signals.](http://www.ncbi.nlm.nih.gov/pubmed/12226665/) Xie Q, et al. Nature, 2002 Sep 12. PMID 12226665. |
| AEE27578 | NAC domain-containing protein 6 [Arabidopsis thaliana] | ANAC006; F21B7.11; NAC domain containing protein 6; NAC006 |  |
| AEC05581 | NAC domain containing protein 35 [Arabidopsis thaliana] | anac034; ANAC035; Arabidopsis NAC domain containing protein 34; LONG VEGETATIVE PHASE 1; LOV1; NAC domain containing protein 35; NAC035 |  |
| Q8H115 | RecName: Full=NAC domain-containing protein 102; Short=ANAC102 | ANAC102; MBK5.27; MBK5_27; NAC domain containing protein 102; NAC102 | [The low-oxygen-induced NAC domain transcription factor ANAC102 affects viability of Arabidopsis seeds following low-oxygen treatment.](http://www.ncbi.nlm.nih.gov/pubmed/19176720/) Christianson JA, et al. Plant Physiol, 2009 Apr. PMID 19176720 |
| Q9ZNU2 | RecName: Full=NAC domain-containing protein 18; Short=ANAC018;  AltName: Full=Protein NO APICAL MERISTEM; Short=AtNAM.  NAM NAC domain-containing protein 18 [ **Arabidopsis thaliana** (thale cress) ] | ANAC018; Arabidopsis NAC domain containing protein 18; ATNAM; F14G24.15; F14G24_15; NAC DOMAIN PROTEIN NAM; NAC-REGULATED SEED MORPHOLOGY 2; NAM; NARS2; NO APICAL MERISTEM | [Molecular characterization of AtNAM: a member of the Arabidopsis NAC domain superfamily.](http://www.ncbi.nlm.nih.gov/pubmed/12175016/) Duval M, et al. Plant Mol Biol, 2002 Sep. PMID 12175016  [NAC family proteins NARS1/NAC2 and NARS2/NAM in the outer integument regulate embryogenesis in Arabidopsis.](http://www.ncbi.nlm.nih.gov/pubmed/18849494/) Kunieda T, et al. Plant Cell, 2008 Oct. PMID 18849494  [SND1, a NAC domain transcription factor, is a key regulator of secondary wall synthesis in fibers of Arabidopsis.](http://www.ncbi.nlm.nih.gov/pubmed/17114348/) Zhong R, et al. Plant Cell, 2006 Nov. PMID 17114348  [Enhanced Y1H assays for Arabidopsis.](http://www.ncbi.nlm.nih.gov/pubmed/22037706/) Gaudinier A, et al. Nat Methods, 2011 Oct 30. PMID 22037706 |
| Q8LGC6 | RecName: Full=Nascent polypeptide-associated complex subunit alpha-like protein 5; Short=NAC-alpha-like protein 5; AltName: Full=Alpha-NAC-like protein 5  NACA5 Nascent polypeptide-associated complex subunit alpha-like protein 5 [ **Arabidopsis thaliana** (thale cress) ] | F9L11.19; F9L11_19; NACA5; nascent polypeptide-associated complex subunit alpha-like protein 5 |  |
| Q9SZY1 | RecName: Full=Nascent polypeptide-associated complex subunit alpha-like protein 4; Short=NAC-alpha-like protein 4; AltName: Full=Alpha-NAC-like protein 4  AT4G10480 Nascent polypeptide-associated complex subunit alpha-like protein 4 [ **Arabidopsis thaliana** (thale cress) ] | F7L13.60; F7L13_60 |  |
| Q6ICZ8 | RecName: Full=Nascent polypeptide-associated complex subunit alpha-like protein 3; Short=NAC-alpha-like protein 3; AltName:  Full=Alpha-NAC-like protein 3. | MAC12.19; MAC12_19; NACA3; nascent polypeptide-associated complex subunit alpha-like protein 3 |  |
| Q94JX9 | RecName: Full=Nascent polypeptide-associated complex subunit alpha-like protein 2; Short=NAC-alpha-like protein 2; AltName:  Full=Alpha-NAC-like protein 2. | NACA2 nascent polypeptide-associated complex subunit alpha-like protein 2 [ **Arabidopsis thaliana** (thale cress) ] |  |
| Q9LHG9 | RecName: Full=Nascent polypeptide-associated complex subunit  alpha-like protein 1; Short=NAC-alpha-like protein 1; AltName:  Full=Alpha-NAC-like protein 1 | AT3G12390 Nascent polypeptide-associated complex subunit alpha-like protein 1 [ **Arabidopsis thaliana** (thale cress) ] |  |
| NP_187845 | Nascent polypeptide-associated complex subunit alpha-like protein 1  [Arabidopsis thaliana]. | AT3G12390 Nascent polypeptide-associated complex subunit alpha-like protein 1 [ **Arabidopsis thaliana** (thale cress) ] |  |
| NP_001078369 | Nascent polypeptide-associated complex subunit alpha-like protein [Arabidopsis thaliana].  AT4G10480 Nascent polypeptide-associated complex subunit alpha-like protein 4 [ **Arabidopsis thaliana** (thale cress) ] | F7L13.60; F7L13_60 |  |
| NP_192786 | Nascent polypeptide-associated complex subunit alpha-like protein 4  [Arabidopsis thaliana].  AT4G10480 Nascent polypeptide-associated complex subunit alpha-like protein 4 [ **Arabidopsis thaliana** (thale cress) ] | F7L13.60; F7L13_60 |  |
| AEE82889 | Nascent polypeptide-associated complex subunit alpha-like protein 4  [Arabidopsis thaliana].  AT4G10480 Nascent polypeptide-associated complex subunit alpha-like protein 4 [ **Arabidopsis thaliana** (thale cress) ] | F7L13.60; F7L13_60 |  |
| AEE82888 | Nascent polypeptide-associated complex subunit alpha-like protein 4  [Arabidopsis thaliana].  AT4G10480 Nascent polypeptide-associated complex subunit alpha-like protein 4 [ **Arabidopsis thaliana** (thale cress) ] | F7L13.60; F7L13_60 |  |
| AEE75192 | Nascent polypeptide-associated complex subunit alpha-like protein 1  [Arabidopsis thaliana]. | AT3G12390 Nascent polypeptide-associated complex subunit alpha-like protein 1 [ ***Arabidopsis thaliana*** (thale cress) ] |  |
| NP_190516 | nascent polypeptide-associated complex subunit alpha-like protein 2  [Arabidopsis thaliana]. | NACA2 nascent polypeptide-associated complex subunit alpha-like protein 2 [ ***Arabidopsis thaliana*** (thale cress) ] |  |
| AEE78547 | nascent polypeptide-associated complex subunit alpha-like protein 2  [Arabidopsis thaliana]. | NACA2 nascent polypeptide-associated complex subunit alpha-like protein 2 [ ***Arabidopsis thaliana*** (thale cress) ] |  |
| NP_196889 | nascent polypeptide-associated complex subunit alpha-like protein 3  [Arabidopsis thaliana]. | MAC12.19; MAC12_19; NACA3; nascent polypeptide-associated complex subunit alpha-like protein 3 |  |
| NP_564415 | Nascent polypeptide-associated complex subunit alpha-like protein 5  [Arabidopsis thaliana] | F9L11.19; F9L11_19; NACA5; nascent polypeptide-associated complex subunit alpha-like protein 5 |  |
| AEE31552 | Nascent polypeptide-associated complex subunit alpha-like protein 5  [Arabidopsis thaliana]. | F9L11.19; F9L11_19; NACA5; nascent polypeptide-associated complex subunit alpha-like protein 5 |  |
| AED91950 | nascent polypeptide-associated complex subunit alpha-like protein 3  [Arabidopsis thaliana]. | MAC12.19; MAC12_19; NACA3; nascent polypeptide-associated complex subunit alpha-like protein 3 |  |
| NP_564238 | suppressor of gamma response 1 [Arabidopsis thaliana] | ANAC008; Arabidopsis NAC domain containing protein 8; F2J7.1; SOG1; SUPPRESSOR OF GAMMA RADIATION 1 | [The role of SOG1, a plant-specific transcriptional regulator, in the DNA damage response.](http://www.ncbi.nlm.nih.gov/pubmed/24736489/) Yoshiyama KO, et al. Plant Signal Behav, 2014. PMID 24736489  [Suppressor of gamma response 1 (SOG1) encodes a putative transcription factor governing multiple responses to DNA damage.](http://www.ncbi.nlm.nih.gov/pubmed/19549833/) Yoshiyama K, et al. Proc Natl Acad Sci U S A, 2009 Aug 4. PMID 19549833  [ATM-mediated phosphorylation of SOG1 is essential for the DNA damage response in Arabidopsis.](http://www.ncbi.nlm.nih.gov/pubmed/23907539/) Yoshiyama KO, et al. EMBO Rep, 2013 Sep. PMID 23907539 |
| NP_177466 | nascent polypeptide-associated complex subunit beta [Arabidopsis  thaliana]. | AT1G73230 nascent polypeptide-associated complex subunit beta [ ***Arabidopsis thaliana*** *(thale cress*) ]  Also known as: T18K17.10; T18K17_10 |  |
| AEE35432 | nascent polypeptide-associated complex subunit beta [Arabidopsis  thaliana]. | AT1G73230 nascent polypeptide-associated complex subunit beta *[* ***Arabidopsis thaliana*** *(thale cress)* ]  T18K17.10; T18K17_10 |  |
| BAB03146 | unnamed protein product [Arabidopsis thaliana]. | AT3G12390 Nascent polypeptide-associated complex subunit alpha-like protein 1 [ ***Arabidopsis thaliana*** *(thale cress) ]* |  |
| Q7DMA9 | RecName: Full=Peptidyl-prolyl cis-trans isomerase PASTICCINO1;  AltName: Full=70 kDa peptidyl-prolyl isomerase; AltName:  Full=FK506-binding protein 72; Short=AtFKBP72; AltName:  Full=Immunophilin FKBP72; AltName: Full=Peptidyl-prolyl cis-trans  isomerase FKBP72; Short=PPIase FKBP72; AltName: Full=Rotamase. | DEI1; PAS1; PASTICCINO 1 | [Mutation in the Arabidopsis PASTICCINO1 gene, which encodes a new FK506-binding protein-like protein, has a dramatic effect on plant development.](http://www.ncbi.nlm.nih.gov/pubmed/9566922/) Vittorioso P, et al. Mol Cell Biol, 1998 May. PMID 9566922  [The C terminus of the immunophilin PASTICCINO1 is required for plant development and for interaction with a NAC-like transcription factor.](http://www.ncbi.nlm.nih.gov/pubmed/16803883/) Smyczynski C, et al. J Biol Chem, 2006 Sep 1. PMID 16803883 |
| NP_179397 | vascular related NAC-domain protein 1 [Arabidopsis thaliana]. | ANAC037; Arabidopsis NAC domain containing protein 37; T27K22.7; T27K22_7; vascular related NAC-domain protein 1; VND1 | [VND-INTERACTING2, a NAC domain transcription factor, negatively regulates xylem vessel formation in Arabidopsis.](http://www.ncbi.nlm.nih.gov/pubmed/20388856/) Yamaguchi M, et al. Plant Cell, 2010 Apr. PMID 20388856 |
| NP_177338 | vascular related NAC-domain protein 7 [Arabidopsis thaliana]. | ANAC030; Arabidopsis NAC domain containing protein 30; F17M19.8; F17M19_8; vascular related NAC-domain protein 7; VND7 | [Verticillium infection triggers VASCULAR-RELATED NAC DOMAIN7-dependent de novo xylem formation and enhances drought tolerance in Arabidopsis.](http://www.ncbi.nlm.nih.gov/pubmed/23023171/) Reusche M, et al. Plant Cell, 2012 Sep. PMID 23023171  [Vascular-related NAC-DOMAIN7 is involved in the differentiation of all types of xylem vessels in Arabidopsis roots and shoots.](http://www.ncbi.nlm.nih.gov/pubmed/18445131/) Yamaguchi M, et al. Plant J, 2008 Aug. PMID 18445131 |
| AAM16178 | AT3g12390/T2E22_130 [Arabidopsis thaliana]. | AT3G12390 Nascent polypeptide-associated complex subunit alpha-like protein 1 *[* ***Arabidopsis thaliana*** *(thale cress)* ] |  |
| AAG51031 | nascent polypeptide associated complex alpha chain, putative;  85450-84199 [Arabidopsis thaliana]. | AT3G12390 Nascent polypeptide-associated complex subunit alpha-like protein 1 *[* ***Arabidopsis thaliana*** *(thale cress) ]* |  |
| AAF31282 | Very similar to alpha-NACs, (Nascent polypeptide > [Arabidopsis  thaliana]. | F9L11.19; F9L11_19; NACA5; nascent polypeptide-associated complex subunit alpha-like protein 5 |  |
| AAL66951 | alpha NAC-like protein [Arabidopsis thaliana]. | alpha NAC-like protein [Arabidopsis thaliana] |  |
| AAD03429 | similar to nascent polypeptide associated complex alpha chain  [Arabidopsis thaliana]. | similar to nascent polypeptide associated complex alpha chain [Arabidopsis thaliana] |  |
| AAL15389 | F9L11.19/F9L11.19 [Arabidopsis thaliana]. | F9L11.19; F9L11_19; NACA5; nascent polypeptide-associated complex subunit alpha-like protein 5 |  |
| AAK82495 | AT3g12390/T2E22_130 [Arabidopsis thaliana] | AT3G12390 Nascent polypeptide-associated complex subunit alpha-like protein 1 [ ***Arabidopsis thaliana*** *(thale cress) ]* | [High heterogeneity within the ribosomal proteins of the Arabidopsis thaliana 80S ribosome.](http://www.ncbi.nlm.nih.gov/pubmed/15821981/) Giavalisco P, et al. Plant Mol Biol, 2005 Mar. PMID 15821981  [Comparative proteomic analysis of NaCl stress-responsive proteins in Arabidopsis roots.](http://www.ncbi.nlm.nih.gov/pubmed/17916636/) Jiang Y, et al. J Exp Bot, 2007. PMID 17916636  [Identification and characterization of nuclear pore complex components in Arabidopsis thaliana.](http://www.ncbi.nlm.nih.gov/pubmed/21189294/) Tamura K, et al. Plant Cell, 2010 Dec. PMID 21189294 |
| AAK74040 | F9L11.19/F9L11.19 [Arabidopsis thaliana]. | F9L11.19; F9L11_19; NACA5; nascent polypeptide-associated complex subunit alpha-like protein 5 |  |
| AAK48972 | alpha NAC-like protein [Arabidopsis thaliana]. | alpha NAC-like protein [Arabidopsis thaliana] |  |
| AAT41858 | At5g13850 [Arabidopsis thaliana]. | MAC12.19; MAC12_19; NACA3; nascent polypeptide-associated complex subunit alpha-like protein 3 | [Comparative proteomic analysis of NaCl stress-responsive proteins in Arabidopsis roots.](http://www.ncbi.nlm.nih.gov/pubmed/17916636/) Jiang Y, et al. J Exp Bot, 2007. PMID 17916636  [High heterogeneity within the ribosomal proteins of the Arabidopsis thaliana 80S ribosome.](http://www.ncbi.nlm.nih.gov/pubmed/15821981/) Giavalisco P, et al. Plant Mol Biol, 2005 Mar. PMID 15821981  [Isolation and proteomic characterization of the Arabidopsis Golgi defines functional and novel components involved in plant cell wall biosynthesis.](http://www.ncbi.nlm.nih.gov/pubmed/22430844/) Parsons HT, et al. Plant Physiol, 2012 May. PMID 22430844 |
| CAB62452 | alpha NAC-like protein [Arabidopsis thaliana]. | NACA2 nascent polypeptide-associated complex subunit alpha-like protein 2 |  |
| NP_197847 | TCV-interacting protein [Arabidopsis thaliana]. | ANAC091; Arabidopsis NAC domain containing protein 91; TCV-interacting protein; TIP | [HRT-mediated hypersensitive response and resistance to Turnip crinkle virus in Arabidopsis does not require the function of TIP, the presumed guardee protein.](http://www.ncbi.nlm.nih.gov/pubmed/18785827/) Jeong RD, et al. Mol Plant Microbe Interact, 2008 Oct. PMID 18785827  [Turnip crinkle virus coat protein inhibits the basal immune response to virus invasion in Arabidopsis by binding to the NAC transcription factor TIP.](http://www.ncbi.nlm.nih.gov/pubmed/24418554/) Donze T, et al. Virology, 2014 Jan 20. PMID 24418554  [HRT gene function requires interaction between a NAC protein and viral capsid protein to confer resistance to turnip crinkle virus.](http://www.ncbi.nlm.nih.gov/pubmed/11041886/) Ren T, et al. Plant Cell, 2000 Oct. PMID 11041886 |
| NP_850986 | protein LONG VEGETATIVE PHASE 1 [Arabidopsis thaliana] | anac034; ANAC035; Arabidopsis NAC domain containing protein 34; LONG VEGETATIVE PHASE 1; LOV1; NAC domain containing protein 35; NAC035 |  |
| AEE75627 | protein CUP-SHAPED COTYLEDON 1 [Arabidopsis thaliana]. | ANAC054; Arabidopsis NAC domain containing protein 54; ATNAC1; CUC1; CUP-SHAPED COTYLEDON1 | [Involvement of CUP-SHAPED COTYLEDON genes in gynoecium and ovule development in Arabidopsis thaliana.](http://www.ncbi.nlm.nih.gov/pubmed/10750709/) Ishida T, et al. Plant Cell Physiol, 2000 Jan. PMID 10750709  [PETAL LOSS is a boundary gene that inhibits growth between developing sepals in Arabidopsis thaliana.](http://www.ncbi.nlm.nih.gov/pubmed/22507233/) Lampugnani ER, et al. Plant J, 2012 Sep. PMID 22507233  [A mechanistic link between STM and CUC1 during Arabidopsis development.](http://www.ncbi.nlm.nih.gov/pubmed/21685178/) Spinelli SV, et al. Plant Physiol, 2011 Aug. PMID 21685178,  [The ENHANCER OF SHOOT REGENERATION 2 gene in Arabidopsis regulates CUP-SHAPED COTYLEDON 1 at the transcriptional level and controls cotyledon development.](http://www.ncbi.nlm.nih.gov/pubmed/17056621/) Ikeda Y, et al. Plant Cell Physiol, 2006 Nov. PMID 17056621  [The NAC domain mediates functional specificity of CUP-SHAPED COTYLEDON proteins.](http://www.ncbi.nlm.nih.gov/pubmed/15500463/) Taoka K, et al. Plant J, 2004 Nov. PMID 15500463 |
| ABL67723 | SND1 [Arabidopsis thaliana]. | ANAC012; F6N18.15; F6N18_15; NAC domain containing protein 12; NAC SECONDARY WALL THICKENING PROMOTING 3; NAC012; NST3; SECONDARY WALL-ASSOCIATED NAC DOMAIN 1; SND1 | [ANAC012, a member of the plant-specific NAC transcription factor family, negatively regulates xylary fiber development in Arabidopsis thaliana.](http://www.ncbi.nlm.nih.gov/pubmed/17565617/) Ko JH, et al. Plant J, 2007 Jun. PMID 17565617  [Arabidopsis VASCULAR-RELATED NAC-DOMAIN6 directly regulates the genes that govern programmed cell death and secondary wall formation during xylem differentiation.](http://www.ncbi.nlm.nih.gov/pubmed/20952636/)  [The MYB46 transcription factor is a direct target of SND1 and regulates secondary wall biosynthesis in Arabidopsis.](http://www.ncbi.nlm.nih.gov/pubmed/17890373/) Zhong R, et al. Plant Cell, 2007 Sep. PMID 17890373, |
| AAP82630 | cup-shaped cotyledon 3 [Arabidopsis thaliana]. | ANAC031; Arabidopsis NAC domain containing protein 31; CUC3; CUP SHAPED COTYLEDON3; F15M4.8; NAC368 | [Arabidopsis CUP-SHAPED COTYLEDON3 regulates postembryonic shoot meristem and organ boundary formation.](http://www.ncbi.nlm.nih.gov/pubmed/17122068/) Hibara K, et al. Plant Cell, 2006 Nov. PMID 17122068, [Free PMC Article](http://www.ncbi.nlm.nih.gov/pmc/articles/PMC1693926/)  [The CUP-SHAPED COTYLEDON3 gene is required for boundary and shoot meristem formation in Arabidopsis.](http://www.ncbi.nlm.nih.gov/pubmed/12837947/) Vroemen CW, et al. Plant Cell, 2003 Jul. PMID 12837947,  [Evolution and diverse roles of the CUP-SHAPED COTYLEDON genes in Arabidopsis leaf development.](http://www.ncbi.nlm.nih.gov/pubmed/21258003/) Hasson A, et al. Plant Cell, 2011 Jan. PMID 21258003  [Cytokinin overproduction-caused alteration of flower development is partially mediated by CUC2 and CUC3 in Arabidopsis.](http://www.ncbi.nlm.nih.gov/pubmed/19913077/) Li XG, et al. Gene, 2010 Jan 15. PMID 19913077  [Interplay of miR164, CUP-SHAPED COTYLEDON genes and LATERAL SUPPRESSOR controls axillary meristem formation in Arabidopsis thaliana.](http://www.ncbi.nlm.nih.gov/pubmed/18346190/) Raman S, et al. Plant J, 2008 Jul. PMID 18346190 |
| [Q6ZI65](http://www.uniprot.org/uniprot/Q6ZI65) | [LOC_Os02g38130](http://rice.plantbiology.msu.edu/cgi-bin/ORF_infopage.cgi?orf=LOC_Os02g38130.1) [Oryza sativa Japonica Group]. | OsNAC19 [ *Oryza sativa Japonica Group* ] |  |
| [Q7F2L3](http://www.uniprot.org/uniref/UniRef100_Q7F2L3) | [LOC_Os05g34830](http://rice.plantbiology.msu.edu/cgi-bin/ORF_infopage.cgi?orf=LOC_Os05g34830.1) [Oryza sativa Japonica Group]. | OsNAC51 [ ***Oryza sativa Japonica Group*** ] |  |
| AIA78390 | [LOC_Os04g52810](http://rice.plantbiology.msu.edu/cgi-bin/ORF_infopage.cgi?orf=LOC_Os04g52810) [Oryza sativa Japonica Group]. | OsNAC45 [*Oryza sativa Japonica Group*] |  |
| AEO53051 | secondary wall NAC transcription factor 6 [Oryza sativa Japonica  Group]. | OsNAC06  *[Oryza sativa Japonica* Group | [The histone deacetylase OsHDAC1 epigenetically regulates the OsNAC6 gene that controls seedling root growth in rice.](http://www.ncbi.nlm.nih.gov/pubmed/19453457/) Chung PJ, et al. Plant J, 2009 Sep. PMID 19453457 |
| AEO53050 | secondary wall NAC transcription factor 5 [Oryza sativa Japonica  Group]. | OsNAC05 |  |
| AFK74453 | [LOC_Os03g61319](http://rice.plantbiology.msu.edu/cgi-bin/ORF_infopage.cgi?orf=LOC_Os03g61319) [Oryza sativa Japonica  Group]. | OsNAC39 |  |
| AFM54252 | [LOC_Os08g33670](http://rice.plantbiology.msu.edu/cgi-bin/ORF_infopage.cgi?orf=LOC_Os08g33670) [Oryza sativa Japonica Group]. | OsNAC83 [Oryza sativa Japonica Group] |  |
| AFL91796 | NAC transcription factor 69 [Oryza sativa Japonica Group]. | ONAC069 [Oryza sativa Japonica Group] |  |
| AFK74450 | [LOC_Os03g59730](http://rice.plantbiology.msu.edu/cgi-bin/ORF_infopage.cgi?orf=LOC_Os03g59730.1)  NAC transcription factor 39 [Oryza sativa Japonica Group]. | OsNAC36 [Oryza sativa Japonica Group] |  |
| Q8H4S4 | RecName: Full=NAC transcription factor ONAC010. | ONAC010 |  |
| [Q8H824](http://www.uniprot.org/uniprot/Q8H824) | [LOC_Os03g12120](http://rice.plantbiology.msu.edu/cgi-bin/ORF_infopage.cgi?orf=LOC_Os03g12120.1) [Oryza sativa Japonica Group] | OsNAC029 |  |
| ABD52007 | stress-induced transcription factor NAC1 [Oryza sativa Japonica  Group]. | OsNAC1 [Oryza sativa Indica Group |  |
| [Q6AVQ6](http://www.uniprot.org/uniref/UniRef100_Q6AVQ6) | LOC_Os03g62470 [Oryza sativa Japonica Group | OsNAC40 |  |
| [B7F0X7](http://www.uniprot.org/uniref/UniRef100_B7F0X7) | [LOC_Os04g40140](http://rice.plantbiology.msu.edu/cgi-bin/ORF_infopage.cgi?orf=LOC_Os04g40140.1) [Oryza sativa Japonica Group | OsNAC43 [ **Oryza sativa Japonica Group** ] |  |
| [PF02365.8](http://pfam.janelia.org/family?acc=PF02365.8) | [LOC_Os11g08210](http://rice.plantbiology.msu.edu/cgi-bin/ORF_infopage.cgi?orf=LOC_Os11g08210), [Os11g0184900](http://rapdb.dna.affrc.go.jp/cgi-bin/gbrowse_details/IRGSP40?name=Os11g0184900;class=locus_id) [Oryza sativa Japonica Group]. | OsNAC112 [ **Oryza sativa Japonica Group** ] |  |
| AFL91783 | [LOC_Os05g35170](http://rice.plantbiology.msu.edu/cgi-bin/ORF_infopage.cgi?orf=LOC_Os05g35170) [Oryza sativa Japonica Group]. | OsNAC52 [ **Oryza sativa Japonica Group** ] |  |
| [PF02365.8](http://pfam.janelia.org/family?acc=PF02365.8) | [LOC_Os01g66120](http://rice.plantbiology.msu.edu/cgi-bin/ORF_infopage.cgi?orf=LOC_Os01g66120.1) | OsNAC11 [ Oryza sativa Japonica Group ] |  |
| B9FGS4 | [LOC_Os05g43960](http://rice.plantbiology.msu.edu/cgi-bin/ORF_infopage.cgi?orf=LOC_Os05g43960) [Oryza sativa Japonica Group]. | OsNAC54 [ **Oryza sativa Japonica Group** ] |  |
| [PF02365.8](http://pfam.janelia.org/family?acc=PF02365.8) | [LOC_Os07g04560](http://rice.plantbiology.msu.edu/cgi-bin/ORF_infopage.cgi?orf=LOC_Os07g04560) [Oryza sativa Japonica Group]. | OsNAC65 [ **Oryza sativa Japonica Group** ] |  |
| [PF02365.8](http://pfam.janelia.org/family?acc=PF02365.8) | LOC_Os08g06140 [Oryza sativa Japonica Group]. | OsNAC80 [ **Oryza sativa Japonica Group** ] |  |
| BAG86835 | [LOC_Os07g48550](http://rice.plantbiology.msu.edu/cgi-bin/ORF_infopage.cgi?orf=LOC_Os07g48550) [Oryza sativa Japonica Group]. | [OsNAC76](http://grassius.org/proteininfo.html?tfid=22208) [ **Oryza sativa Japonica Group** ] |  |
| [PF02365.8](http://pfam.janelia.org/family?acc=PF02365.8) | [LOC_Os07g48450](http://rice.plantbiology.msu.edu/cgi-bin/ORF_infopage.cgi?orf=LOC_Os07g48450) , [Os07g0683200](http://rapdb.dna.affrc.go.jp/cgi-bin/gbrowse_details/IRGSP40?name=Os07g0683200;class=locus_id) [Oryza sativa Japonica Group]. | OsNAC75 [ **Oryza sativa Japonica Group** ] |  |
| [PF02365.8](http://pfam.janelia.org/family?acc=PF02365.8) | [LOC_Os11g03300](http://rice.plantbiology.msu.edu/cgi-bin/ORF_infopage.cgi?orf=LOC_Os11g03300), [Os11g0126900](http://rapdb.dna.affrc.go.jp/cgi-bin/gbrowse_details/IRGSP40?name=Os11g0126900;class=locus_id)  [Oryza sativa Japonica Group]. | [OsNAC107](http://grassius.org/proteininfo.html?tfid=22401) [ **Oryza sativa Japonica Group** ] |  |
| [PF02365.8](http://pfam.janelia.org/family?acc=PF02365.8) | [LOC_Os12g03040](http://rice.plantbiology.msu.edu/cgi-bin/ORF_infopage.cgi?orf=LOC_Os12g03040), [Os12g0123700](http://rapdb.dna.affrc.go.jp/cgi-bin/gbrowse_details/IRGSP40?name=Os12g0123700;class=locus_id) [Oryza sativa Japonica Group]. | OsNAC118 [ **Oryza sativa Japonica Group** |  |
| BAF26797 | [LOC_Os10g38834](http://rice.plantbiology.msu.edu/cgi-bin/ORF_infopage.cgi?orf=LOC_Os10g38834) [Oryza sativa Japonica Group]. | OsNAC105 [ **Oryza sativa Japonica Group** ] |  |
| [PF02365.8](http://pfam.janelia.org/family?acc=PF02365.8) | [LOC_Os12g43530](http://rice.plantbiology.msu.edu/cgi-bin/ORF_infopage.cgi?orf=LOC_Os12g43530) [Oryza sativa Japonica Group] | OsNAC127 [ **Oryza sativa Japonica Group** ] |  |
| [PF02365.8](http://pfam.janelia.org/family?acc=PF02365.8) | [LOC_Os12g41680](http://rice.plantbiology.msu.edu/cgi-bin/ORF_infopage.cgi?orf=LOC_Os12g41680) [Oryza sativa Japonica Group] | [OsNAC126](http://grassius.org/proteininfo.html?tfid=22084) [ ***Oryza sativa Japonica Group*** ] |  |
| [PF02365.8](http://pfam.janelia.org/family?acc=PF02365.8) | [LOC_Os06g15690](http://rice.plantbiology.msu.edu/cgi-bin/ORF_infopage.cgi?orf=LOC_Os06g15690) [Oryza sativa Japonica Group] | OsNAC59 [ ***Oryza sativa Japonica Group*** ] |  |
| [PF02365.8](http://pfam.janelia.org/family?acc=PF02365.8) | [LOC_Os06g46270](http://rice.plantbiology.msu.edu/cgi-bin/ORF_infopage.cgi?orf=LOC_Os06g46270) [Oryza sativa Japonica Group] | [OsNAC63](http://grassius.org/proteininfo.html?tfid=23293) [ ***Oryza sativa Japonica Group*** *]* |  |
| [PF02365.8](http://pfam.janelia.org/family?acc=PF02365.8) | [LOC_Os03g60080](http://rice.plantbiology.msu.edu/cgi-bin/ORF_infopage.cgi?orf=LOC_Os03g60080.1) [Oryza sativa Japonica Group] | OsNAC37 [ ***Oryza sativa Japonica Group*** ] |  |
| [PF02365.8](http://pfam.janelia.org/family?acc=PF02365.8) | [LOC_Os12g29330](http://rice.plantbiology.msu.edu/cgi-bin/ORF_infopage.cgi?orf=LOC_Os12g29330) [Oryza sativa Japonica Group] | [OsNAC125](http://grassius.org/proteininfo.html?tfid=22934) *[* ***Oryza sativa Japonica Group*** ] | [The Rice Annotation Project Database (RAP-DB): 2008 update.](http://www.ncbi.nlm.nih.gov/pubmed/18089549/) Rice Annotation Project, et al. Nucleic Acids Res, 2008 Jan. PMID 18089549.  [Curated genome annotation of Oryza sativa ssp. japonica and comparative genome analysis with Arabidopsis thaliana.](http://www.ncbi.nlm.nih.gov/pubmed/17210932/) Rice Annotation Project, et al. Genome Res, 2007 Feb. PMID 17210932, |
| [PF02365.8](http://pfam.janelia.org/family?acc=PF02365.8) | [LOC_Os02g34970](http://rice.plantbiology.msu.edu/cgi-bin/ORF_infopage.cgi?orf=LOC_Os02g34970.1) | OsNAC17 |  |
