# Supplementary material for: The essence of NAC gene family to the cultivation of drought-resistant soybean (Glycine max L. Merr.) cultivars
Source: BMC Plant Biol. 2017 Feb 28;17:55. doi: 10.1186/s12870-017-1001-y (PMC5330122; doi:10.1186/s12870-017-1001-y)
Supplement: Additional file 3: — Table of motifs information. (DOCX 14 kb) [file 12870_2017_1001_MOESM3_ESM.docx]

Motifs information

| MOTIF | WIDTH | BEST POSSIBLE MATCH |
| --- | --- | --- |
| 1 | 41 | GEKEWYFFCPRDRKYPNGTRTNRATAAGYWKATGKDKPIYS |
| 2 | 18 | HLPPGFRFHPTDEELVVY |
| 3 | 15 | PKGEKTNWIMHEYRL |
| 4 | 21 | VDIIAEVDLYKCEPWDLPEKA |
| 5 | 15 | LIGMKKTLVFYKGRA |
| 6 | 15 | QDEWVLCRVFKKSNT |
| 7 | 50 | IDEFIPTLEGEQGICYTHPEKLPGVKKDGSIRHFFHRPSKAYTTGTRKRR |
| 8 | 11 | YLKRKVAGQPF |
| 9 | 29 | NNQACDQVTDWRALDKFVASQLSQEAAHC |
| 10 | 21 | HCGLDGGHVRWHKTGKTRPVI |
| 11 | 50 | DGGNDDYSNSPNFEVVEEIKVNHGMFVSTRQVVDTFFHQIVPSQTVQVHL |
| 12 | 50 | IMVNQGHSFFRKFKAYVMGKLIKPSKTIASAIVFIFALVLMHCVYLKEQV |
| 13 | 29 | DQIWDCGAYRECPNCHHHIDNQDVAHEWP |
| 14 | 29 | SPCWYDDQVSFMQDLDSPKQSCQPNYPYQ |
| 15 | 50 | TFSSASFYSAQQAHQIGNSHFPDYFYTHQEQSMLRMLCENHGYCAGQRMQ |
| 16 | 21 | CICHDHFMQLPQLESPKLALA |
| 17 | 29 | YPPSKVDIALECARMQHRFVMPPLEVQDF |
| 18 | 39 | WEDPNARTIEIGDVDDGFKTERMVENLRWVGMSSEDMEK |
| 19 | 50 | QVTEDDKHQPMIPAHSEEAISNIITPGDIHSDGCDACDAQNQIVEPVAEE |
| 20 | 31 | PMEHERDDSMDDMIGGIPPSINVGHMSARFH |
