# Supplementary figures and images for: The essence of NAC gene family to the cultivation of drought-resistant soybean (Glycine max L. Merr.) cultivars
Source: BMC Plant Biol. 2017 Feb 28;17:55. doi: 10.1186/s12870-017-1001-y (PMC5330122; doi:10.1186/s12870-017-1001-y)

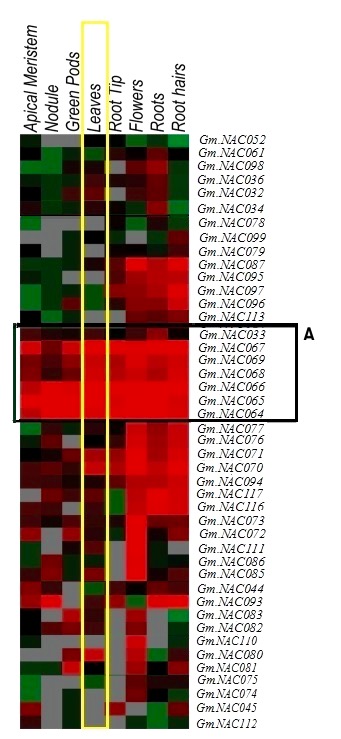

Supplement: Additional file 4: — Heat map representation for tissue-specific expression of 44 predicted stress-responsive and 6 previously reported dehydration-responsive GmNAC genes. Expression patterns of 44 GmNAC genes were analysed using Illumina transcriptome data (source: [22]). Box A indicates a group of ubiquitously expressed GmNAC genes in the eight types of tissues examined. The colors indicate expression intensity (red, high expression; green, low expression; grey, no expression). (JPG 74 kb) [file 12870_2017_1001_MOESM4_ESM.jpg]

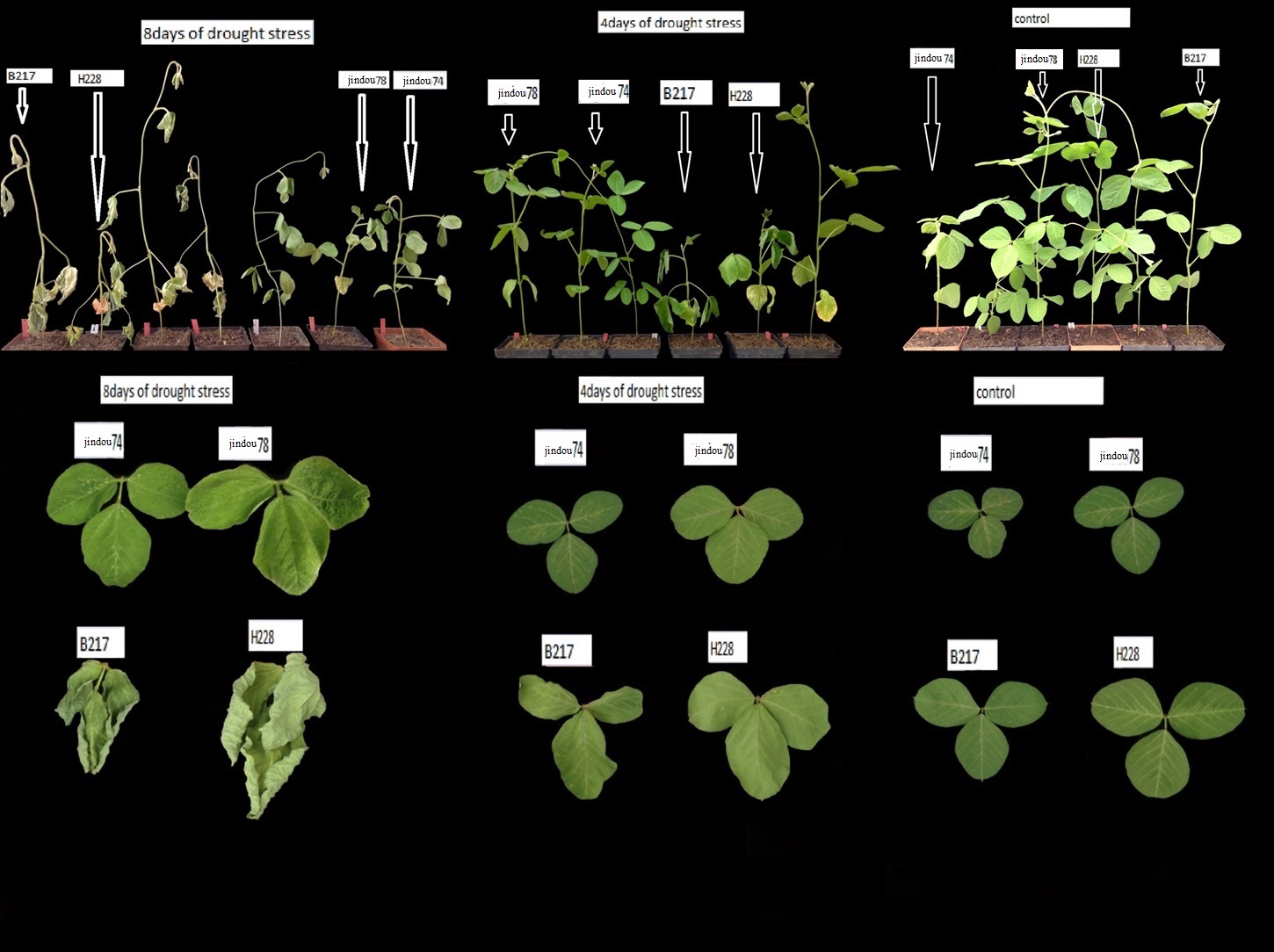

Supplement: Additional file 6 — Phenotypes of drought at different times of drought treatments for four selected varieties: drought tolerant (Jindou 74 and 78) and sensitive (H228, B217) varieties in (a) and (b) as follow: (a) whole plants of: control, 4 days of drought treatments, and 8 days of drought treatments. (b) The leaves of control, 4 days, 8 days of drought treatments. (JPG 265 kb) [file 12870_2017_1001_MOESM6_ESM.jpg]
