# Supplementary material for: The essence of NAC gene family to the cultivation of drought-resistant soybean (Glycine max L. Merr.) cultivars
Source: BMC Plant Biol. 2017 Feb 28;17:55. doi: 10.1186/s12870-017-1001-y (PMC5330122; doi:10.1186/s12870-017-1001-y)
Supplement: Additional file 5: — Table of specific primers information. (DOCX 21 kb) [file 12870_2017_1001_MOESM5_ESM.docx]

| *GmNAC064* | Glyma.14G152700 |
| --- | --- |
| Glyma.14G152700.1-F | CTGGATCATGCACGAGTATC (Sense) |
| Glyma.14G152700.1-R | CGGTGGTGGTAGTTGTTG (AntiSense) |
| *GmNAC067* | Glyma.04G249000 |
| Glyma.04G249000.1-F | CAGGGAAAGCTCCGAAAG (Sense) |
| Glyma.04G249000.1-R | CGTGCCCTTCTTGTTGT (AntiSense) |
| *GmNAC070* | Glyma.13G279900 |
| Glyma.13G279900.1-F | GGCCACCATTTCTCTCTTC (Sense) |
| Glyma.13G279900.1-R | CGTTCGGGTATTTCCTGTC (AntiSense) |
| *GmNAC005* | Glyma.10G219600 |
| Glyma.10G219600.1-F | CCCAAGTGCATCAGAAGG (Sense) |
| Glyma.10G219600.1-R | CCAGAGAGCAGAGGAGAA (AntiSense) |
| *GmNAC072* | Glyma.12G149100 |
| Glyma.12G149100.1-F | GCTTGCCTCCTGGATTTAG (Sense) |
| Glyma.12G149100.1-R | CTCCAAACGCTGCCTTAC (AntiSense) |
| *GmNAC080* | Glyma.13G280000 |
| Glyma.13G280000.1-F | CCAACCATGACTCACTCTTC (Sense) |
| Glyma.13G280000.1-R | CATCCCTCACAACACTTCC (AntiSense) |
| *GmNAC020* | Glyma.17G154100 |
| Glyma.17G154100.1-F | GCCTTGTGCCGAGTTATT (Sense) |
| Glyma.17G154100.1-R | CAGATGACTTGCTTGAGAGG (AntiSense) |
| *GmNAC117* | Glyma.08G173400 |
| Glyma.08G173400.1-F | CACACAAAGAGACCGTAAGTAT (Sense) |
| Glyma.08G173400.1-R | GTGCCCTTCCTTGATAGAAC (AntiSense) |
| *Gm.NAC040* | Glyma.14G189300 |
| Glyma.14G189300.1-F | CAAACGGTGATGATGTGGA (Sense) |
| Glyma.14G189300.1-R | CTCGGAGTTCTCGGAAATAAC (AntiSense) |
| *Gm.NAC041* | Glyma.02G222300 |
| Glyma.02G222300.1-F | CTTGGGACTTGCCTGATTT (Sense) |
| Glyma.02G222300.1-R | GACTTAATCCTCCGATCCTTTC (AntiSense) |
| *Gm.NAC042* | Glyma.06G138100 |
| Glyma.06G138100.1-F | GGCTACACATGGCTCAAATA (Sense) |
| Glyma.06G138100.1-R | TGTCTTCGGCAGTTCATTC (AntiSense) |
| *Gm.NAC043* | Glyma.04G226700 |
| Glyma.04G226700.1-F | GGCTACACATGGCTCAAATA (Sense) |
| Glyma.04G226700.1-R | TGTCTTCTGCAGTTCATTCC (AntiSense) |
| *Gm.NAC068* | Glyma.06G157400 |
| Glyma.06G157400.1-F | GCGCTAGGGATCAAGAAAG (Sense) |
| Glyma.06G157400.1-R | CCCAATCATCAAGCCTCAA (AntiSense) |
| *Gm.NAC069* | Glyma.04G208300 |
| Glyma.04G208300.1-F | GGAAGCGGCTATTGGAAA (Sense) |
| Glyma.04G208300.1-R | CATTGGCGAGGCGATATT (AntiSense) |
| *Gm.NAC084* | Glyma.19G108800 |
| Glyma.19G108800.1-F | CTTGCCCGTTTCCATCAT (Sense) |
| Glyma.19G108800.1-R | TTGGCCTTGCACCATTAG (AntiSense) |
| *Gm.NAC085* | Glyma.16G043200 |
| Glyma.16G043200.1-F | ACCCTTCAGTCTCCAACA (Sense) |
| Glyma.16G043200.1-R | CCATTGTGGGTTCTCGTATT (AntiSense) |
| *Gm.NAC086* | Glyma.16G151500 |
| Glyma.16G151500.1-F | CAAGCAGTGGTAGAAGAAGAG (Sense) |
| Glyma.16G151500.1-R | TGGCATCCAAGAGATTTGAG (AntiSense) |
| *Gm.NAC087* | Glyma.02G070000 |
| Glyma.02G070000.1-F | AGGAAGAAGGTTGCCTCTAT (Sense) |
| Glyma.02G070000.1-R | CTTGCTCCATTTGGGTACTT (AntiSense) |
| *GmNAC065* | Glyma.13G030900 |
| Glyma.13G030900.1-F | **GAATGGCTTTGTACGGAGAG (Sense)** |
| Glyma.13G030900.1-R | **CCCAACAGGTTTCGGTTT (AntiSense)** Hairpin Blast |
| *GmNAC066* | Glyma.06G114000 |
| Glyma.06G114000.1-F | **GCAGGGAAAGCTCCTAAAG (Sense)** |
| Glyma.06G114000.1-R | **ATCGTGCCCTTCTTGTTG (AntiSense)** |
| *GmNAC071* | Glyma.12G221500 |
| Glyma.12G221500.1-F | **CAGCCCTAGAGACAGGAAATA (Sense)** |
| Glyma.12G221500.1-R | **GGGCTTTGCCAATGTAGAA (AntiSense)** |
| *GmNAC073* | Glyma.06G248900 |
| Glyma.06G248900.1-F | **GTTTGCCTCCTGGGTTTAG (Sense)** |
| Glyma.06G248900.1-R | **CTCTCCAAACACTGCCTTAC (AntiSense)** |
| *GmNAC116* | **Glyma.15G254000** |
| **Glyma.15G254000**.1-F | **GGGCACCCAAAGGAAATAA (Sense)** |
| **Glyma.15G254000**.1-R | **CAGCTACCCATTCTAGGTTTG (AntiSense)** |
| *GmNAC081* | Glyma.12G221400 |
| Glyma.12G221400.1-F | **CACATGAGTGCGAGGTTT (Sense)** |
| Glyma.12G221400.1-R | **AGGCGTAGTAGTAGCAGTAG (AntiSense)** |
| *GmNAC082* | Glyma.12G148900 |
| Glyma.12G148900.1-F | **GGTGGTTATAGGCAATGGTAG (Sense)** |
| Glyma.12G148900.1-R | **TTTGGAGGCCGAATTAGAAG (AntiSense)** |
| *GmNAC083* | Glyma.06G249100 |
| Glyma.06G249100.1-F | **GCTGGATGATTGGGTGTTAT (Sense)** |
| Glyma.06G249100.1-R | **GTAGCTCGTGGACATCTTTG (AntiSense)** |
| *GmNAC004* | Glyma.20G172100 |
| Glyma.20G172100.1-F | **CTGGTTCCGCTGATTGTT (Sense)** Hairpin Blast |
| Glyma.20G172100.1-R | **CCTCGGGATCCCACTTAT (AntiSense)** Hairpin Blast |
| *GmNAC021* | Glyma.05G113000 |
| Glyma.05G113000.1-F | **TGGAAAGCCACTGGTAAAG (Sense)** Hairpin Blast |
| Glyma.05G113000.1-R | **GCCAAGATCATCGCAGAG (AntiSense)** Hairpin Blast |
