# Supplementary material for: The essence of NAC gene family to the cultivation of drought-resistant soybean (Glycine max L. Merr.) cultivars
Source: BMC Plant Biol. 2017 Feb 28;17:55. doi: 10.1186/s12870-017-1001-y (PMC5330122; doi:10.1186/s12870-017-1001-y)
Supplement: Additional file 7: — The data of 139 GmNACs of Fig. 3. (source: 66 K Affymetrix Soybean Array GeneChip of Dung Tien Le et al. [41]. (DOCX 22 kb) [file 12870_2017_1001_MOESM7_ESM.docx]

| gene ID | V6D | R2D | V6-D/V6-C | R2-D/R2-C |
| --- | --- | --- | --- | --- |
| *Gm.NAC001* | 1.1078335 | 1.95475 | 1.108 | 1.955 |
| *Gm.NAC002* | 1.0005726 | 1.12223 | 1.001 | -1.122 |
| *Gm.NAC003* | 1.1851047 | 1.25960 | 1.185 | 1.260 |
| *Gm.NAC004* | 1.2603965 | N/A | 1.26 | 1.639 |
| *Gm.NAC005* | 1.9278811 | 1.88766 | 1.928 | 1.888 |
| *Gm.NAC006* | 1.1689013 | 1.23771 | 1.169 | 1.238 |
| *Gm.NAC007* | 1.7029246 | 1.11401 | -1.703 | -1.114 |
| *Gm.NAC009* | 1.4149258 | 5.85405 | -1.415 | -5.854 |
| *Gm.NAC010* | 1.0607259 | N/A | -1.061 | -1.073 |
| *Gm.NAC011* | 1.0181692 | 1.22642 | 1.018 | 1.226 |
| *Gm.NAC012* | 1.3384155 | 1.20166 | -1.338 | -1.202 |
| *Gm.NAC013* | 1.0388081 | 1.08385 | 1.039 | 1.084 |
| *Gm.NAC014* | 1.4550496 | 1.01263 | -1.455 | -1.013 |
| *Gm.NAC015* | 1.1440384 | 1.02179 | -1.144 | 1.022 |
| *Gm.NAC016* | 1.3384155 | 1.20166 | -1.338 | -1.202 |
| *Gm.NAC017* | 1.3117629 | 1.21751 | -1.312 | 1.218 |
| *Gm.NAC020* | 1.0624385 | 1.31581 | 1.062 | 1.316 |
| *Gm.NAC021* | 1.1622523 | 1.50222 | 1.162 | 1.502 |
| *Gm.NAC022* | 1.1068983 | 4.86286 | 1.107 | 4.863 |
| *Gm.NAC023* | 1.702959 | 3.73489 | 1.703 | 3.748 |
| *Gm.NAC024* | 1.0459307 | 1.82447 | 1.046 | 1.824 |
| *Gm.NAC026* | 1.1744235 | 1.47636 | 1.174 | -1.476 |
| *Gm.NAC028* | 1.1180112 | 1.41779 | -1.118 | 1.418 |
| *Gm.NAC029* | 1.0321443 | 1.56247 | 1.032 | 1.562 |
| *Gm.NAC030* | 1.3134868 | 1.64323 | -1.313 | 1.643 |
| *Gm.NAC031* | 1.5565869 | 1.78804 | -1.557 | 1.788 |
| *Gm.NAC032* | 1.3057666 | 1.10179 | -1.306 | 1.102 |
| *Gm.NAC033* | 1.0701199 | 1.08671 | -1.07 | 1.087 |
| *Gm.NAC034* | 1.0272659 | 1.57696 | 1.027 | -1.577 |
| *Gm.NAC035* | 1.0817542 | 1.87116 | -1.082 | -1.871 |
| *Gm.NAC036* | 1.1561372 | 1.10134 | 1.156 | 1.101 |
| *Gm.NAC038* | 1.5930243 | 1.14984 | 1.593 | -1.150 |
| *Gm.NAC039* | 1.0589577 | 1.22603 | -1.059 | -1.226 |
| *Gm.NAC040* | 2.4137273 | 3.68973 | 2.414 | 3.690 |
| *Gm.NAC041* | 2.966464 | 3.28571 | 2.966 | 3.286 |
| *Gm.NAC042* | 4.4586573 | 1.18699 | 4.459 | 1.187 |
| *Gm.NAC043* | 2.373883 | 1.05270 | 2.374 | -1.053 |
| *Gm.NAC044* | 1.18652 | 1.24013 | -1.187 | -1.240 |
| *Gm.NAC045* | 1.3675724 | 2.05633 | -1.368 | 2.056 |
| *Gm.NAC047* | 1.1421275 | 6.36386 | -1.739 | 6.364 |
| *Gm.NAC048* | 1.7390049 | 2.71037 | -1.946 | 2.710 |
| *Gm.NAC049* | 1.9460397 | 1.02616 | -1.946 | -1.026 |
| *Gm.NAC050* | 4.5951467 | 1.39702 | -4.595 | -1.397 |
| *Gm.NAC051* | 1.4138596 | 1.71615 | -1.414 | 1.716 |
| *Gm.NAC052* | 3.1355927 | 2.09596 | -3.136 | -2.096 |
| *Gm.NAC053* | 2.1285686 | 1.57267 | -2.129 | -1.573 |
| *Gm.NAC054* | 1.649996 | 2.17274 | -1.65 | 2.173 |
| *Gm.NAC055* | 1.7581934 | 2.61429 | -1.758 | 2.614 |
| *Gm.NAC056* | 1.0035472 | 1.42380 | 1.004 | 1.424 |
| *Gm.NAC057* | 1.103101 | 1.68429 | 1.103 | 1.684 |
| *Gm.NAC058* | 1.0675362 | 1.15781 | -1.068 | 1.158 |
| *Gm.NAC059* | 1.0157281 | 1.04080 | 1.016 | -1.041 |
| *Gm.NAC060* | 1.025803 | 1.23356 | 1.026 | -1.234 |
| *Gm.NAC061* | 1.2818646 | 1.13432 | -1.282 | 1.134 |
| *Gm.NAC064* | 3.390187 | 3.10694 | 3.39 | 3.107 |
| *Gm.NAC065* | 4.7662215 | 2.08528 | 4.766 | 2.085 |
| *Gm.NAC066* | 5.2484365 | 1.93028 | 5.248 | 1.930 |
| *Gm.NAC067* | 1.617604 | 1.96899 | 1.618 | 1.969 |
| *Gm.NAC068* | 4.4975696 | 1.40150 | 4.498 | -1.402 |
| *Gm.NAC069* | 4.9553547 | 1.05956 | 4.955 | 1.060 |
| *Gm.NAC070* | 27.688866 | 21.14616 | 27.689 | 21.146 |
| *Gm.NAC071* | 45.244545 | 17.90063 | 45.245 | 17.901 |
| *Gm.NAC072* | 10.982247 | 26.64531 | 10.982 | 26.645 |
| *Gm.NAC073* | 11.225643 | 11.04213 | 11.226 | 11.042 |
| *Gm.NAC074* | 3.9006605 | 1.23437 | 3.901 | -1.234 |
| *Gm.NAC075* | 2.891784 | 1.01785 | 2.892 | -1.018 |
| *Gm.NAC076* | 1.7145462 | 3.26063 | -1.715 | 3.261 |
| *Gm.NAC077* | 1.3770089 | 2.28606 | 1.377 | 2.286 |
| *Gm.NAC078* | 1.5905147 | 1.00167 | -1.591 | 1.002 |
| *Gm.NAC079* | 1.1124517 | 1.66377 | -1.112 | 1.664 |
| *Gm.NAC080* | 7.0080895 | 2.50883 | -7.008 | 2.509 |
| *Gm.NAC081* | 1.2410139 | 2.00439 | -1.241 | 2.004 |
| *Gm.NAC082* | 1.9968342 | 4.41153 | 1.997 | 4.412 |
| *Gm.NAC083* | 1.0349767 | 3.41716 | -1.035 | 3.417 |
| *Gm.NAC085* | 1.6155622 | 1.03553 | -1.616 | 1.036 |
| *Gm.NAC086* | 4.1175175 | 19.64366 | 4.118 | 19.644 |
| *Gm.NAC087* | 2.7169437 | 23.25397 | 2.717 | 23.254 |
| *Gm.NAC088* | 1.0368184 | 1.38122 | 1.037 | 1.381 |
| *Gm.NAC090* | 1.117896 | 1.14483 | -1.118 | 1.145 |
| *Gm.NAC093* | 5.5475597 | 2.93054 | 5.548 | 2.931 |
| *Gm.NAC094* | 1.2846161 | 1.49722 | -1.285 | 1.497 |
| *Gm.NAC095* | 1.0504904 | 1.23062 | 1.05 | 1.231 |
| *Gm.NAC096* | 1.6817365 | 1.37882 | 1.682 | 1.379 |
| *Gm.NAC097* | 1.2930706 | 1.12070 | 1.293 | 1.121 |
| *Gm.NAC098* | 1.2256663 | 1.08126 | 1.226 | 1.081 |
| *Gm.NAC099* | 1.4929551 | 1.01372 | -1.493 | 1.014 |
| *Gm.NAC100* | 1.0437711 | 1.32536 | -1.328 | -1.325 |
| *Gm.NAC101* | 1.1661137 | 1.17332 | -1.166 | 1.173 |
| *Gm.NAC102* | 1.108236 | 1.00822 | -1.108 | -1.008 |
| *Gm.NAC103* | 1.0969425 | 1.09939 | -1.097 | -1.099 |
| *Gm.NAC104* | 1.5006006 | 1.31433 | -1.501 | 1.314 |
| *Gm.NAC105* | 1.1107602 | 1.04772 | -1.111 | -1.048 |
| *Gm.NAC106* | 1.746198 | 1.73503 | 1.746 | -1.735 |
| *Gm.NAC107* | 1.350002 | 1.37266 | 1.35 | -1.373 |
| *Gm.NAC108* | 1.1580119 | 1.02324 | -1.158 | -1.023 |
| *Gm.NAC109* | 1.1452705 | 1.07611 | -1.145 | 1.076 |
| *Gm.NAC110* | 1.0238683 | 1.08440 | 1.024 | 1.084 |
| *Gm.NAC111* | 1.7101953 | 1.50932 | 1.71 | 1.509 |
| *Gm.NAC112* | 1.3858691 | 1.15843 | 1.386 | 1.158 |
| *Gm.NAC113* | 1.0071895 | 1.07663 | 1.007 | -1.077 |
| *Gm.NAC116* | 1.2323719 | 4.21841 | 1.232 | 4.218 |
| *Gm.NAC117* | 1.1207099 | 5.52506 | 1.121 | 5.525 |
| *Gm.NAC120* | 1.1619706 | 1.12356 | -1.162 | 1.124 |
| *Gm.NAC121* | 1.0494354 | 1.06022 | -1.049 | -1.060 |
| *Gm.NAC122* | 1.3446734 | 1.06644 | -1.345 | 1.066 |
| *Gm.NAC123* | 1.1487329 | 1.20186 | 1.149 | -1.202 |
| *Gm.NAC124* | 1.0143039 | 1.55566 | 1.014 | -1.556 |
| *Gm.NAC125* | 1.1536003 | 2.28992 | 1.154 | -2.290 |
| *Gm.NAC126* | 1.1315871 | 1.12097 | -1.132 | 1.121 |
| *Gm.NAC127* | 1.0418563 | 1.09386 | 1.042 | 1.094 |
| *Gm.NAC129* | 1.0838829 | 1.44766 | -1.084 | -1.448 |
| *Gm.NAC130* | 1.1274844 | 1.19454 | -1.127 | -1.195 |
| *Gm.NAC131* | 1.626234 | 1.28036 | 1.626 | -1.280 |
| *Gm.NAC132* | 1.0785235 | 1.10404 | -1.079 | -1.104 |
| *Gm.NAC133* | 1.0975708 | 1.15650 | -1.098 | 1.156 |
| *Gm.NAC134* | 1.2151623 | 1.11641 | -1.215 | -1.116 |
| *Gm.NAC135* | 1.0003349 | 1.05601 | -1 | 1.056 |
| *Gm.NAC136* | 1.0001514 | 1.12814 | 1 | 1.128 |
| *Gm.NAC137* | 1.0369589 | 1.04674 | -1.037 | 1.047 |
| *Gm.NAC138* | 3.9621694 | 2.08852 | 3.962 | 2.089 |
| *Gm.NAC139* | 1.6633667 | 2.32286 | 1.663 | 2.323 |
